# Expert consensus on the clinical application of PI3K/AKT/mTOR inhibitors in the treatment of advanced breast cancer

**DOI:** 10.1002/cai2.10

**Published:** 2022-06-21

**Authors:** The Society of Clinical Research of Oncology Medications of China Anticancer Association, Breast Cancer Expert Committee of National Cancer Quality Control Center, Boao Cancer Innovation Institute

**Keywords:** breast cancer, PI3K/AKT/mTOR inhibitors

## Abstract

Phosphoinositide 3‐kinase (PI3K)/protein kinase B (PKB or AKT)/mammalian target of rapamycin (mTOR) signaling pathway (PAM pathway) plays an important role in the development of breast cancer and are closely associated with the resistance to endocrine therapy in advanced breast cancer. Therefore, anticancer treatment targeting key molecules in this signaling pathway has become a research hotspot in recent years. Randomized clinical trials have demonstrated that PI3K/AKT/mTOR inhibitors bring significant clinical benefit to patients with advanced breast cancer, especially to those with hormone receptor (HR)‐positive, human epidermal growth factor receptor (HER) 2‐negative advanced breast cancer. Alpelisib, a PI3K inhibitor, and everolimus, an mTOR inhibitor, have been approved by FDA. Based on their high efficacy and relatively good safety profile, an expanded indication of everolimus in breast cancer has been approved by National Medical Products Administration (NMPA). Alpelisib is expected to be approved in China in the near future. The members of the consensus expert panel reached this consensus to comprehensively define the role of PI3K/AKT/mTOR signaling pathway in breast cancer, efficacy and clinical applications of PI3K/AKT/mTOR inhibitors, management of adverse reactions, and PIK3CA mutation detection, to promote the understanding of PI3K/AKT/mTOR inhibitors for Chinese oncologists, improve clinical decision‐making, and prolong the survival of target patient population.

## Graphical abstract

This consensus comprehensively defines the role of PI3K/AKT/mTOR signaling pathway in breast cancer, efficacy and clinical applications of PI3K/AKT/mTOR inhibitors, management of adverse reactions, and PIK3CA mutation detection, to promote the understanding of PI3K/AKT/mTOR inhibitors for Chinese oncologists, improve clinical decision‐making, and prolong the survival of target patient population

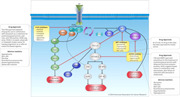

**Abbreviations**
AI: Aromatase inhibitor
BCRP: Breast cancer resistance protein
BSA: Body surface area
CBR: Clinical benefit rate
CDK4/6: Dysregulation of the cyclin D‐cyclin‐dependent kinase4/6
ctDNA: Circulating tumor DNA
CYP2C9: Cytochrome P450 2C9
CYP3A4: Cytochrome P450 3A4
ddPCR: Droplet digital PCR
EM: Erythema multiforme
ERα: Estrogen receptor α
ESMO: European Society for Medical Oncology
FDA: Food and Drug Administration
FPG: Fasting plasma glucose
HbA1c: Glycosylated hemoglobin
HER2: Human epidermal growth factor receptor 2
HR: Hormone receptor
NCCN: National Comprehensive Cancer Network
NF‐κB: Nuclear factor kappa‐B
NGS: Next generation sequencing
NMPA: National Medical Products Administration
ORR: Objective response rate
OS: Overall survival
P‐gp: P‐glycoprotein
p70S6K: p70 ribosomal protein S6 kinase
PFS: Progression‐free survival
PI3K/AKT/mTOR: Phosphoinositide 3‐kinase/protein kinase B/mammalian target of rapamycin
PIK3CA: Phosphatidylinositol‐4,5‐bisphosphate 3‐kinase
PIP3: Phosphatidylinositol‐3,4,5‐triphosphate
PJP: Pneumocystis pneumonia
PTEN: Phosphatase and tensin homologue deleted on chromosome 10
RT‐PCR: Reverse Transcription Polymerase Chain Reaction
RTK: Receptor tyrosine kinase
SABCS: San Antonio Breast Cancer Symposium
SGLT‐2: Sodium‐dependent glucose transporters 2
SJS: Stevens‐Johnson syndrome
TEN: Toxic epidermal necrolysis
ULN: Upper Limit of Normal

## 1 INTRODUCTION

Among all malignancies, breast cancer has the highest incidence and fifth highest mortality rate worldwide in the female population [1]. According to GLOBOCAN 2020, the global cancer data, there were 2.261 million new cases and 685 thousand deaths in 2020 [1], while in China, 416 thousand new cases and 117 thousand deaths were reported in 2020 [2]. Breast cancer is considered a heterogeneous group of diseases at the molecular level, whose comprehensive care regimen depends on the molecular subtype of each individual patient, and approximately 70% of all breast cancer cases are hormone receptor (HR)‐positive [3, 4]. Although endocrine therapy, chemotherapy, and targeted therapy (such as CDK4/6 inhibitors and anti‐HER2 therapy) have significantly decreased the risk of recurrence, most patients still end up with drug resistance, resulting in disease progression. Consequently, there is an urgent need to explore the mechanisms of resistance and develop an effective strategy to overcome drug resistance [5, 6].

Phosphoinositide 3‐kinase (PI3K)/protein kinase B (PKB or AKT)/mammalian target of rapamycin (mTOR) signaling pathway (PAM pathway) is involved in the regulation of tumor cell growth, proliferation, survival, angiogenesis, and so forth, which is important in the pathogenesis and progression of breast cancer [6, 7, 8, 9, 10]. Meanwhile, normalizing the overactivation of the PAM pathway is also one of the major mechanisms of action in endocrine therapy, chemotherapy, and targeted therapy. Over the course of treatment, not only do the breast cancer cells adapt to estrogen deprivation and develop resistance to chemotherapy via multidrug resistance‐associated proteins as well as the antiapoptotic mechanisms, but proteins associated with the PAM pathway also contribute to the resistance to CDK4/6 inhibitors and anti‐HER2 treatment [5, 6, 7, 11, 12].

Several PAM‐specific‐targeted therapies are either already available or under research, and PAM pathway inhibitors can be categorized into three major classes: (1) PI3K inhibitors, such as alpelisib, taselisib, and inavolisib; (2) AKT inhibitors, such as capivasertib and ipatasertib; and (3) mTOR inhibitors, such as everolimus [5, 13]. Alpelisib is the first PI3K inhibitor that has been approved for breast cancer, and the combination therapy of alpelisib and fulvestrant has been approved by the United States Food and Drug Administration (FDA) for male and postmenopausal female patients with PIK3CA (the gene encoding p110α, the PI3K catalytic subunit)‐mutated, HR‐positive/HER2‐negative advanced breast cancer who suffer from disease progression during or after endocrine therapy [5, 11, 14]. In HR‐positive/HER2‐negative breast cancer, PIK3CA is one of the most commonly mutated genes, and is also more prevalent among patients in China than in western countries [15]. Everolimus in combination with exemestane has also been approved by the FDA and China's National Medical Products Administration, in postmenopausal patients with HR‐positive/HER2‐negative advanced breast cancer who failed letrozole or anastrozole therapy [16]. Multiple phase II/III trials have confirmed that in terms of progression‐free survival, patients with endocrine‐resistant, HR‐positive/HER2‐negative advanced breast cancer can benefit from combination therapy of everolimus plus endocrine therapy (such as letrozole, exemestane, or fulvestrant) [17, 18, 19, 20, 21]. According to a multicenter, retrospective study in China, in patients with HR‐positive/HER2‐negative advanced breast cancer who exhibited disease progression after palbociclib therapy, a CDK4/6 inhibitor, an increase of up to 5.1 months in median PFS was observed after receiving everolimus in combination with endocrine therapy [22]. In addition, AKT inhibitors are still under research, but none of them has been approved as of now.

To further promote clinical studies on PAM‐targeted drugs and provide standardized management of their clinical application and safe practice, a consensus on the clinical application of PAM‐specific therapy has been reached, with collaborated efforts of experts brought together by the Society of Clinical Research of Oncology Medications of China Anticancer Association, Breast Cancer Expert Committee of National Cancer Quality Control Center, the Society of Onco‐Pathology of China Anticancer Association, and Boao Cancer Innovation Institute. This consensus will focus on currently available PAM inhibitors both in China and abroad, summarizing their mechanisms of action, profiles of clinical benefit and adverse reactions, as well as key points on the detection of PIK3CA mutation, to guide their clinical application, efficacy monitoring, and management of adverse effects of these medications.

## 2 PAM PATHWAY MEDIATES THE PATHOGENESIS AND PROGRESSION OF BREAST CANCER AND DRUG RESISTANCE

### 2.1 The role of PAM pathway in the pathogenesis and progression of breast cancer

**Figure 1:**
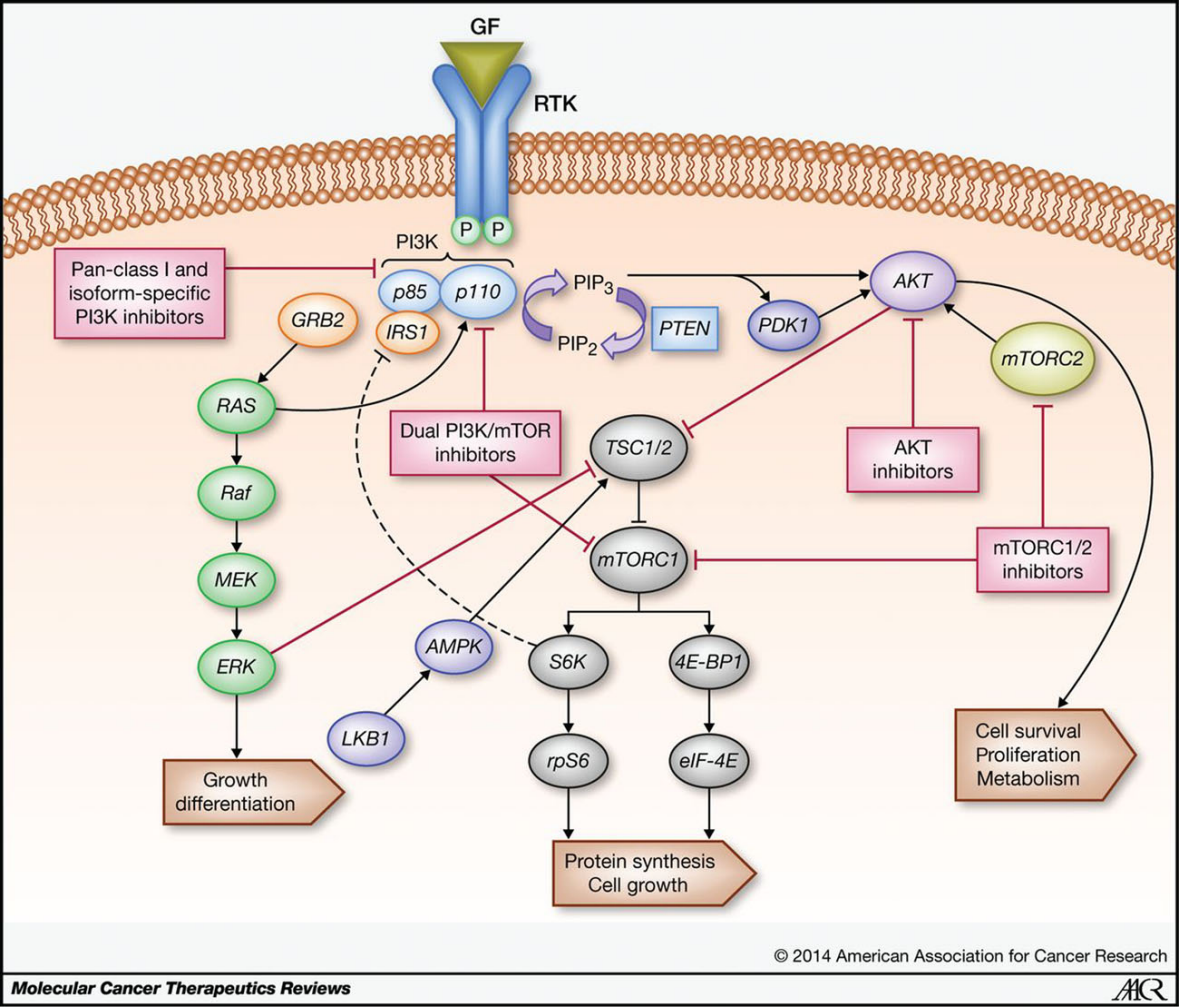
PAM signaling pathway and mechanisms of action of PAM pathway inhibitors [10].

Activation of receptor tyrosine kinase (RTK) stimulates the PI3K complex (p85 and p110), which converts phosphatidylinositol 4,5‐bisphosphate to phosphatidylinositol 3,4,5‐trisphosphate (PIP3). Then, PIP3 phosphorylates and activates AKT, resulting in the activation of the mTOR complex after a series of phosphorylations [8, 10, 23]. In breast cancer, PAM is the most commonly activated pathway, which is closely related to tumor pathogenesis, progression, and metastasis [7, 8, 9, 10]. The PI3K complex functions the upstream of PAM pathway and there are three classes of PI3K. Class I is the major class of PI3K that drives tumor pathogenesis [24]. Studies have demonstrated that the gain‐of‐function mutation in PIK3CA (the gene encoding p110α, the catalytic subunit) is associated with the overactivation of PI3K, which is an effective mechanism of oncogenesis, especially in breast cancer [5, 24]. Meanwhile, AKT is one of the major effector molecules downstream, and once activated, phosphorylates many target molecules in the nucleus and cytosol, which then activates a large number of downstream substrates that regulates cell proliferation, contributing to tumor cell growth, proliferation, and angiogenesis [7, 23, 25]. Furthermore, AKT facilitates survival and inhibits apoptosis of tumor cells by inhibiting BAD and BAX, members of the Bcl‐2 family, which is a group of proapoptotic genes, and downregulating forkhead transcription factors like FOXO. A downstream target of PI3K/AKT is mTOR, activation of which results in phosphorylation of ribosomal protein S6 kinase (S6K) and eukaryotic translation initiation factor 4E (eIF4E)‐binding protein 1 (4EBP‐1), permitting the transcription, proliferation, growth, and protein synthesis of tumor cells [7, 9, 24, 26, 27] (Figure 1).

### 2.2 The role of PAM pathway in the treatment of resistant breast cancer

#### 2.2.1 Resistance to endocrine therapy

A potential mechanism of resistance to endocrine therapy is the overactivation of PAM pathway. Preclinical data have shown that PI3K and AKT can phosphorylate the Ser167 locus of ERα, independently activating ERα in the absence of estrogen. Therefore, the interaction between estrogen receptor (ER) and the overactivation of PAM pathway enables breast cancer cells to adapt to estrogen deprivation, resulting in desensitization of breast cancer cells to endocrine therapy [5, 7]. During the development of resistance to endocrine therapy, after prolonged estrogen deprivation, the main activation targets for PI3K include MCF‐7 and p70S6 kinase, the substrate of mTOR in MDA‐MB‐361 cells. Meanwhile, PIK3CA mutation and PTEN loss trigger estrogen‐independent growth of breast cancer cells. In PAM pathway, PIK3CA is the most common mutated gene, detected in almost 50% of ER‐positive breast cancer patients [5, 24]. In addition, upregulation of RTK, which causes enhanced activation of the downstream PAM pathway, is also involved in the mechanisms of resistance [5].

#### 2.2.2 Resistance to CDK4/6 inhibitors

Additionally, PAM pathway is also involved in the acquired resistance to CDK4/6 inhibitors. In a model of acquired resistance to palbociclib monotherapy, compared to parental cell lines, resistant cells underwent significant changes in PAM pathway‐associated proteins (such as upregulation of pAKT^S473^, pAKT^th308^, and P70S6K, as well as downregulation of PTEN), including 25 upregulated and 18 downregulated proteins. By means of pathway enrichment analysis, several changes in cancer‐associated signaling pathways have been identified, including PI3K, mTOR, AMPK, and apoptosis induction pathways [12]. Among the various mechanisms mediating drug resistance to CDK4/6 inhibitors found in HR‐positive/HER2‐negative breast cancer cells, upregulation of the PI3K/mTOR pathway is a very common one, making it a potential target for reversal of drug resistance [12]. Furthermore, in the case of acquired resistance, palbociclib is unable to inhibit the Rb signal, and thus loses control of tumor cell growth, while targeted PI3K therapy can block PI3K/AKT/mTOR pathway, thus effectively inhibiting the proliferation of resistant cells [12].

#### 2.2.3 Resistance to anti‐HER2 therapies

As a member of the receptor tyrosine kinase ErbB family, HER2 forms heterodimers with other members of this family, triggering self‐phosphorylation of the receptor tyrosine kinase domain and activating downstream pathways, including PAM pathway. Aberrant activation of PAM pathway is closely associated with resistance to anti‐HER2 therapies [5, 28]. The most common variation of HER2 receptor molecule is a truncated form of HER2 (p95‐HER2), and overexpression of p95‐HER2 can induce resistance to anti‐HER2 therapies via the activation of the PI3K/AKT signaling pathway [5]. Plus, trastuzumab, an anti‐HER2 agent, facilitates the expression of HER3 in breast cancer cells, and its overexpression can also activate PI3K/AKT pathway, resulting in resistance to anti‐HER2 therapies [5]. Other activating factors of PAM pathway, including PIK3CA mutation, AKT1 mutation, AKT2 amplification, and loss of cancer suppressor gene PTEN, can also cause resistance to anti‐HER2 agents, such as trastuzumab and lapatinib [5, 28].

Extensive preclinical evidence has shown that PAM inhibitors can overcome the resistance to anti‐HER2 therapies. Copanlisib, a PI3K inhibitor has been proved capable of sensitizing anti‐HER2 therapy‐resistant cells, potentiating the effects of trastuzumab and lapatinib. The growth of breast tumor can also be inhibited, to a great extent, by the combination of alpelisib and anti‐HER2 monoclonal antibodies [5].

#### 2.2.4 Resistance to chemotherapy

Transcription of breast cancer resistance protein (BCRP) and expression of P‐glycoprotein (P‐gp) are facilitated by PAM pathway via the KEAP1‐Nrf2 axis and NF‐κB pathway, with the exact mechanisms still unclear. The efflux of chemotherapy agents is increased by BCRP and P‐gp, resulting in chemoresistance [5]. The phosphorylation of AKT is also proved to be positively correlated to the activity, migration, and apoptosis of breast cancer cells, potentially leading to chemoresistance [29].

The efficacy of PAM pathway inhibitors in combination with chemotherapy in patients with chemoresistant breast cancer is currently under research in many clinical studies. For example, the efficacy of paclitaxel in combination with PI3K inhibitors (pictilisib and buparlisib) has been assessed in two randomized controlled trials, PEGGY (NCT01740336) and BELLE‐4 (NCT01572727). Nonetheless, the median PFS is only slightly prolonged in both studies without significant benefit. Other PI3K inhibitors are also under investigation, such as the application of alpelisib in combination with albumin‐bound paclitaxel in advanced triple‐negative breast cancer with PIK3CA mutation or PTEN loss [5]. Patients with inoperable, locally triple‐negative advanced or metastatic breast cancer were included in another phase II LOTUS study (NCT02162719) and were divided into two groups: one treated with paclitaxel and ipatasertib, an AKT inhibitor, and the other treated with paclitaxel and placebo. Improved PFS was observed in the group treated with paclitaxel combined with iparasertib (6.2 vs. 4.9 months, *p* = 0.037), providing evidence for targeted treatment with AKT inhibitors in patients with triple‐negative breast cancer [5].

### 2.3 The role of PAM pathway inhibitors in the treatment of breast cancer

Up till now, a variety of PAM pathway inhibitors are either already available or under research. Targeted PI3K inhibitors include alpelisib (BYL719), taselisib (GDC‐0032), and inavolisib (GDC‐0077), and alpelisib is the first oral PI3K inhibitor specifically targeting the p110α subtype that has been approved for the treatment of breast cancer [5]. Iparasertib (GDC‐0068) and capivasertib (AZD‐5363) are both AKT inhibitors currently under clinical research, but no AKT inhibitor has yet been approved either in China or abroad [5]. Everolimus is the first mTOR inhibitor approved for the treatment of HR‐positive/HER2‐negative breast cancer [5].

## 3 INSTRUCTIONS FOR CLINICAL APPLICATION

### 3.1 The efficacy of PAM pathway inhibitors

#### 3.1.1 PI3K inhibitors

##### Alpelisib

Patients with HR‐positive/HER2‐negative advanced breast cancer who had received endocrine therapy were enrolled in the SOLAR‐1 trial [30], and the efficacy and safety of fulvestrant and alpelisib combination therapy were assessed in comparison with fulvestrant and placebo. The results showed that in patients with PIK3CA mutation, PFS was significantly prolonged (11.0 vs. 5.7 months) and the risk of disease progression or death was decreased by 35% in patients treated with combined alpelisib and fulvestrant treatment, compared to fulvestrant monotherapy. Objective response rate (ORR), clinical benefit rate (CBR), and tumor shrinkage rate were all higher in the group treated with alpelisib and fulvestrant than in the group treated with fulvestrant and placebo. Meanwhile, regardless of whether the patient had received CDK4/6 inhibitors before, they could benefit from combination therapy of alpelisib and fulvestrant either as a first‐ or second‐line therapy [31]. According to an update from ESMO in 2020, [32] the median overall survival in the combination therapy group was prolonged by 7.9 months (39.3 vs. 31.4 months) compared to the other group, further confirming the efficacy of this regimen. For HR‐positive/HER2‐negative advanced breast cancer patients with PIK3CA mutation who had received CDK4/6 inhibitor treatment, it is proved by the BYLieve study [33] for the first time that they can benefit from alpelisib treatment in combination with aromatiase inhibitors (AI), with the efficacy and safety profile unaffected by alpelisib. Thus, alpelisib has been confirmed as an important backup option for patients with CDK4/6 inhibitor‐resistant breast cancer [33, 34, 35, 36]. For premenopausal female patients with HR‐positive/HER2‐negative advanced breast cancer, alpelisib in combination with endocrine therapy has been shown in the B‐YOND study [37] to be a potentially effective regimen (with a median PFS of 25.2 months and an ORR of 50.0%). Compared to patients without PIK3CA mutation, more tumor shrinkage was observed in those with PIK3CA mutation after treatment with alpelisib.

Based on these results, it is recommended in the NCCN guidelines [38] that for postmenopausal HR‐positive/HER2‐negative breast cancer patients with PIK3CA mutation, the second‐line treatment of choice should be a combination therapy of alpelisib and fulvestrant (category 1). The ABC6 guidelines [39] also recommend that for patients with PIK3CA (exon 9 or 20) mutation and appropriate HbA1c level who has been exposed to AI, alpelisib combined with fulvestrant could be a treatment option. For ER‐positive/HER2‐negative metastatic breast cancer, alpelisib should only be administered in patients with PIK3CA mutation. Similarly, according to the *Guidelines and Standard of Diagnosis and Treatment of Breast Cancer* by China Anticancer Association [40], in the second‐line treatment of advanced breast cancer, there is evidence supporting the combination therapy of alpelisib, the PI3Kα inhibitor, and endocrine therapy in patients with PI3Kα mutation (detected by ctDNA testing of tumor tissue or peripheral blood) (Table 1).

**Table 1:** Advances in the treatment of advanced breast cancer with alpelisib

Study	Phase	Treatment	Patients	Outcomes
SOLAR‐1 [30, 32]	III	Fulvestrant + alpelisib/placebo	Patients with HR‐positive/HER2‐negative advanced breast cancer who exhibited disease progression during or after endocrine therapy‐based treatment (*n* = 572)	For patients with PIK3CA mutation (treatment group vs. control group):
				Median PFS: 11.0 months versus 5.7 months (HR: 0.65; *p* < .0.001)
				Median OS: 39.3 months versus 31.4 months (HR: 0.86; *p* = 0.15)
BYLieve [33, 34, 36]	II	Alpelisib + fulvestrant or letrozole	HR‐positive/HER2‐negative breast cancer patients with PIK3CA mutation whose previous line of therapy was CDK4/6 inhibitor combined with endocrine therapy and who exhibited disease progression with systemic chemotherapy or endocrine therapy (*n* = 336)	For patients who previously received CDK4/6 inhibitor + AI treatment (Cohort A):
				Progression‐free rate at 6 months: 50.4%. Median PFS: 7.3 months
				For patients who previously received CDK4/6 inhibitor + fulvestrant treatment (Cohort B):
				Progression‐free rate at 6 months: 46.1%. Median PFS: 5.7 months
				For patients who previously received chemotherapy or endocrine therapy after progression with AI (Cohort C):
				Progression‐free rate at 6 months: 48.7%. Median PFS: 5.6 months
B‐YOND [37]	Ib	Alpelisib or buparlisib combined with tamoxifen + goserelin	Premenopausal patients with HR‐positive/HER2‐negative advanced breast cancer who had never received endocrine therapy (*n* = 39)	Patients who received alpelisib treatment:
				Median PFS: 25.2 months
				ORR: 50.0%
				CBR: 56%

Abbreviations: AI, aromatase inhibitor; CBR, clinical benefit rate; HR, hazard ratio; PFS, progression‐free survival; ORR, objective response rate; OS, overall survival.

##### Inavolisib (GDC‐0077)

It was reported at the 2020 SABCS conference that a phase I/Ib clinical study [41] explored the efficacy and safety of inavolisib combined with palbociclib and fulvestrant in HR‐positive/HER2‐negative metastatic breast cancer patients with PIK3CA mutation. This study found that inavolisib at 9 mg, which was the recommended dose in phase II clinical study, in combination with palbociclib and fulvestrant at standard doses, exhibited controllable safety profiles and some antitumor activity. In addition, a pilot study of correlation analysis between clinical outcomes and PIK3CA mutation status in patients with HR‐positive/HER2‐negative metastatic breast cancer treated with inavolisib was also presented at this conference [42], suggesting that a higher ORR was observed in patients with multiple PIK3CA mutations detected in baseline ctDNA testing, compared to those with single PIK3CA mutation. Results from Cohort D were reported at the 2021 SABCS conference [43], revealing a median PFS of 7.1 months and a CBR of 48%. Currently, a phase III clinical study on the efficacy of inavolisib in combination with palbociclib and fulvestrant (NCT04191499) is still underway.

#### 3.1.2 AKT inhibitors

##### Ipatasertib

The IPATunity130 study is an international, multicenter, randomized, placebo‐controlled, double‐blind phase III clinical trial, designed to assess the efficacy and safety of ipatasertib in combination with paclitaxel as first‐line treatment in triple‐negative or HR‐positive/HER2‐negative breast cancer patients with PIK3CA/AKT1/PTEN mutation. The main findings of its Cohort A were presented at the 2020 SABCS conference [44], and no significant difference in PFS was found between the combination therapy group and the paclitaxel monotherapy group in patients with PIK3CA/AKT1/PTEN mutation (median PFS: 7.4 vs. 6.1 months), unable to achieve its goal in terms of the primary endpoint.

##### Capivasertib

The FAKTION trial [45] is a Phase II clinical trial, designed to assess the efficacy and safety of capivasertib in postmenopausal patients with HR‐positive/HER2‐negative metastatic breast cancer who suffered from disease progression after AI therapy, with results showing that PFS was significantly improved by capivasertib (10.3 vs. 4.8 months; hazard ratio [HR] = 0.58; *p* = 0.0018), compared to placebo. It is revealed in further analysis that even higher efficacy was achieved by capivasertib in patients without PI3K mutations (HR = 0.56; 95% confidence interval [CI]: 0.33–0.96; *p* = 0.035). Another study was published in *Clinical Cancer Research* in 2020, which compared capivasertib monotherapy and combination therapy of capivasertib plus fulvestrant in ER‐positive metastatic breast cancer with AKT1 mutation [46]. An ORR of 20% was achieved in the monotherapy group, while in the combination therapy group, for patients who had previously received fulvestrant therapy, the ORR was 36%, but for those who had never received fulvestrant before, the ORR was 20%. Therefore, capivasertib is a potential option for the treatment of ER‐positive metastatic breast cancer.

#### 3.1.3 mTOR inhibitors

##### Everolimus

It has been demonstrated in the BOLERO‐2 [17, 47], PrE0102 [18], and GINECO trials [48] that compared to endocrine therapy alone, everolimus combined with endocrine therapy can prolong PFS in postmenopausal patients with HR‐positive/HER2‐negative advanced breast cancer who had experienced treatment failure with endocrine therapy, showing a median PFS of 7.8–10.3 months and a decrease in the risk of disease progression by 39%–55% (Table 2). The BOLERO‐5 trial [19] was designed to assess the efficacy of everolimus and exemestane combination therapy in postmenopausal ER‐positive/HER2‐negative advanced breast cancer in China. Similar to the BOLERO‐2 trial, an international multicenter trial, a significantly prolonged PFS (median PFS: 7.4 months) was observed in the BOLERO‐5 trial, further supporting the application of everolimus and exemestane combination therapy in postmenopausal patients with ER‐positive/HER2‐negative advanced breast cancer in China (Table 2). The BOLERO‐4 trial [20] demonstrated that a median PFS of 22 months was achieved by combination therapy of everolimus and letrozole as the first‐line treatment in postmenopausal patients with HR‐positive/HER2‐negative advanced breast cancer (Table 2). The MIRACLE trial [21] also demonstrated that PFS was significantly prolonged by combination therapy of everolimus and endocrine therapy, compared to endocrine therapy alone (19.4 vs. 12.9 months) in premenopausal patients with HR‐positive/HER2‐negative advanced breast cancer who had experienced treatment failure with endocrine therapy (Table 2). Furthermore, the TRINITI‐1 trial, a single‐center, retrospective study in the United States, and a multicenter, retrospective study in China all confirmed that in terms of PFS, benefits could be also achieved with combination therapy of everolimus and endocrine therapy with or without CDK4/6 inhibitors in patients with HR‐positive/HER2‐negative advanced or metastatic breast cancer who had experienced disease progression with CDK4/6 inhibitors [22, 49, 50] (Table 3). The multicenter, retrospective study in China found that in terms of PFS, combination therapy of everolimus and endocrine therapy provided benefits for patients with HR‐positive/HER2‐negative metastatic breast cancer who had experienced disease progression with CDK4/6 inhibitors [22] (Table 3).

**Table 2:** Phase II/III trials on combination therapy of everolimus plus endocrine therapy in HR‐positive/HER2‐negative advanced breast cancer.

Postmenopausal patients with HR‐positive/HER2‐negative advanced breast cancer who had experienced treatment failure with endocrine therapy						
Study	Number of cases	Treatment regimen	Median PFS (months)	PFS hazard ratio (HR)	Median OS (months)	OS hazard ratio (HR)
BOLERO‐2 [17, 47]	724	Everolimus + exemestane; exemestane	7.8; 3.2	0.45	31.0; 26.6	0.89
PrE0102 [18]	131	Everolimus + fulvestrant; fulvestrant	10.3; 5.1	0.61	28.3; 31.4	1.31
GINECO [48]	111	Everolimus + tamoxifen; tamoxifen	8.6; 4.5	0.54	Unreached; 32.9	0.45
BOLERO‐5 [19]	159	Everolimus + exemestane; exemestane	7.4; 2.0	0.52	Unreached	Unreached

As first‐line treatment in postmenopausal patients with HR‐positive/HER2‐negative advanced breast cancer					
Study	Number of cases	Treatment regimen	Median PFS (months)	PFS hazard ratio (HR)	Overall survival rate
BOLERO‐4 [20]	200	First‐line treatment: everolimus + letrozole	22	‐	Unreached; overall survival rate at 24 months: 78.8%

Premenopausal patients with HR‐positive/HER2‐negative advanced breast cancer who had experienced treatment failure with endocrine therapy						
Study	Number of cases	Treatment regimen	Median PFS (months)	PFS hazard ratio (HR)	Median OS (months)	OS hazard ratio (HR)
MIRACLE [21]	199	Everolimus + letrozole; letrozole	19.4; 12.9	0.64	Unreached	‐

Abbreviations: HER2, human epidermal growth factor receptor 2; HR, hormone receptor; OS, overall survival; PFS, progression‐free survival.

**Table 3:** Clinical trials and real‐world studies on combination therapy of everolimus plus endocrine therapy with or without CDK4/6 inhibitors in CDK4/6 inhibitor‐resistant, HR‐positive/HER2‐negative advanced or metastatic breast cancer.

Phase I/II clinical trials in patients with HR‐positive/HER2‐negative advanced breast cancer who had experienced disease progression with CDK4/6 inhibitor treatment				
Study	Number of cases	Treatment regimen		CBR at 24 weeks (%)
TRINITI‐1 [49]	104	Exemestane + ribociclib + everolimus		41.1

Real‐world studies in patients with HR‐positive/HER2‐negative metastatic breast cancer who had experienced disease progression with palbociclib treatment, a CDK4/6 inhibitor				
Study	Number of cases	Treatment regimen		Median PFS (months)
Single‐center, retrospective study in the United States [50]	200	Everolimus + exemestane		4.9
Multicenter, retrospective study in China [22]	200	Everolimus + exemestane/fulvestrant/letrozole/anastrozole/toremifene		5.1
		Palbociclib + fulvestrant/anastrozole/exemestane/toremifene		3.1
		Chidamide + exemestane/tamoxifen/fulvestrant/anastrozole		2.6

Abbreviations: CBR, clinical benefit rate; HER2, human epidermal growth factor receptor 2; HR, hormone receptor; PFS, progression‐free survival.

Subgroup analyses in both the BOLERO‐2 trial [17] and the MIRACLE trial [21] reported that the PFS benefit brought on by combination therapy of everolimus and endocrine therapy was not affected by factors, such as age (<65 vs. ≥65 years), presence or absence of visceral metastasis, presence or absence of bone metastasis, primary or secondary resistance to endocrine therapy, presence or absence of recurrence during adjuvant therapy, and so forth.

##### 3.1.3.1 Sirolimus

A retrospective study [51] has shown that for patients with HR‐positive advanced breast cancer, similar median PFS can be achieved with either combination therapy of sirolimus and endocrine therapy or combination therapy of everolimus and endocrine therapy (4.9 and 5.5 months, respectively; HR = 1.56; *p* = 0.142), indicating that sirolimus could be an alternative to everolimus. However, till now, there has been no large‐scale, prospective clinical evidence on this, and sirolimus has not yet been approved for the treatment of breast cancer

### 3.2 Indicated population

#### 3.2.1 PI3K inhibitors (alpelisib)

According to the FDA label, a combination of alpelisib and fulvestrant can be added to an endocrine therapy‐based regimen in the treatment of male and postmenopausal female patients with PIK3CA‐mutated, HR‐positive/HER2‐negative advanced or metastatic breast cancer who exhibited disease progression during or after treatment [14]. According to the drug label in Hong Kong, combination therapy of alpelisib and fulvestant can be administered in postmenopausal female patients with PIK3CA‐mutated, HR‐positive/HER2‐negative locally advanced or metastatic breast cancer who exhibited disease progression after endocrine monotherapy [52]. As for now, no indication has been approved in mainland China for alpelisib.

Expert panel recommendations: Based on the findings of SOLAR‐1 and BYLieve studies, combination therapy of alpelisib and fulvestrant may be used in patients with PIK3CA‐mutated, HR‐positive/HER2‐negative breast cancer who experienced disease progression during or after endocrine therapy‐based regimens. For those with PIK3CA mutation who experienced disease progression with CDK4/6 inhibitors, a combination of alpelisib and endocrine therapy (AI or fulvestrant) is also a preferred option.

#### 3.2.2 mTOR inhibitors (everolimus)

In light of information on the drug label and new indications approved by NMPA, everolimus and exemestane combination therapy can be used in postmenopausal patients with HR‐positive/HER2‐negative advanced breast cancer after treatment failure with letrozole or anastrozole therapy [16].

Expert panel recommendations: With combination therapy of CDK4/6 inhibitors plus endocrine therapy becoming the first‐line treatment of HR‐positive/HER2‐negative advanced breast cancer now, in general, CDK4/6 inhibitor plus fulvestrant is also administered in patients who failed AI therapy. Based on the findings of BOLERO‐2 and BOLERO‐5, as well as real‐world studies, everolimus and exemestane combination therapy may be used in patients with HR‐positive/HER2‐negative advanced breast cancer after treatment failure with endocrine monotherapy or combined therapy. Thus, everolimus and exemestane combination therapy is a very valuable option for cases resistant to CDK4/6 inhibitors but without PIK3CA mutation. Moreover, combination therapies of everolimus plus fulvestrant or tamoxifen have also been proved feasible by PrE0102 and GINECO trials in postmenopausal patients with HR‐positive/HER2‐negative advanced breast cancer after treatment failure with endocrine therapy.

#### 3.2.3 AKT inhibitors

As of now, no drug in this class has been approved for breast cancer.

### 3.3 Drug information

Based on the FDA drug label, drug information about alpelisib [14] and everolimus [16] is summarized in Table 4.

**Table 4:** Pharmacokinetics, dosage, and administration of alpelisib and everolimus

Generic name	Brand name	Dosage form	Manufacturer	Time to maximum plasma concentration (h)	Half‐life (h)	Initial dosage and administration	Dose modification	Route of administration
Alpelisib [14]	Piqray®	Tablets	Novartis	2‐4	8‐9	Swallow tablets whole, 300 mg each time (2 tablets, 150 mg each), once daily, continuous administration^a^^,^ b ^,^ c	Modify dose according to the safety parameters and tolerance of each individual. May discontinue and/or decrease dosage	Take orally with food
Everolimus [16]	Afinitor ®	Tablets	Novartis	1‐2	30	Swallow tablets whole, 10 mg each time, once daily, continuous administration^d^^,^ e	Modify dose according to the safety parameters and tolerance of each individual. May discontinue and/or decrease dosage	Take orally with food or on empty stomach

^a^ Swallow tablets whole. Tablets should not be chewed, crushed or split before swallowing. No tablet should be ingested if it is broken, cracked, or otherwise not intact.

^b^ If a dose of alpelisib is missed, it can be taken with food within 9 h after the time it is usually taken. After more than 9 h, skip the dose for that day. The next day, take alpelisib at the usual time. If the patient vomits after taking the dose, advise the patient not to take an additional dose on that day, and to resume the dosing schedule the next day at the usual time.

^c^ When combined with fulvestrant, the recommended dose of fulvestrant is 500 mg administered on Days 1, 15, and 29, and once monthly thereafter.

^d^ Swallow tablets as is. Tablets should not be chewed or crushed before swallowing, and should be administered orally at the same time each day. Take everolimus once a day, with or without food. Swallow the tablets whole with a glass of water.

^e^ Combined with exemestane.

### 3.4 Drug interactions

#### 3.4.1 PI3K inhibitors (alpelisib)

When taken with strong CYP3A4 inducers, the concentration of alpelisib will be decreased, and thus the effects diminished. Such coadministration should be avoided [15]. Coadministration of alpelisib with a breast cancer resistance protein inhibitor may increase alpelisib concentration, heightening the risk of toxicities. Such coadministration should also be avoided [14]. Coadministration of alpelisib with CYP2C9 substrates can decrease the plasma concentration of these substrates, diminishing their effects. Thus, the plasma concentration of these drugs should be monitored in the case of coadministration [14]. See Table 5 for details about the classification of coadministered drugs and precautions for alpelisib.

**Table 5:** Precautions for coadministration of alpelisib with other drugs [14]

Classification	Drug names	Precautions for alpelisib
CYP3A4 inducers	Including but not limited to rifampin	Coadministration with a strong CYP3A4 inducer may decrease the concentration of alpelisib, diminishing its activity. Such coadministration should be avoided.
BCRP inhibitors	Including but not limited to cyclosporine	Coadministration with BCRP inhibitors may increase the concentration of alpelisib, increasing the risk of toxicities. For patients on alpelisib, BCRP inhibitors should be avoided. If no alternative regimen is available and alpelisib must be coadministered with BCRP inhibitors, adverse effects should be closely monitored due to the risk of increased toxicity.
CYP2C9 substrates	Including but not limited to warfarin	Coadministration with CYP2C9 substrates may decrease the plasma concentration of these substrates, diminishing their activity. Therefore, when coadministered, the concentration of the CYP2C9 substrates should be closely monitored.

Abbreviation: BCRP, breast cancer resistance protein.

#### 3.4.2 mTOR inhibitors (everolimus)

Everolimus is the substrate of CYP3A4 and P‐glycoprotein (PgP), a multidrug efflux pump, which might interact with CYP3A4 and/or PgP inhibitors and inducers [16, 53]. The metabolism of everolimus is slowed down by CYP3A4 and/or PgP inhibitors, raising its plasma concentration, and thus increasing its adverse effects. Therefore, coadministration of everolimus with moderate and strong inhibitors of CYP3A4 and/or PgP inhibitors should be avoided, and under the circumstances where they must be coadministered, the dose of everolimus should be lowered [16, 53]. On the contrary, CYP3A4 and/or PgP inducers accelerate the metabolism of everolism, lowering its plasma concentration, thus diminishing the effects of everolimus. Therefore, coadministration of everolimus and strong CYP3A4 and/or PgP inducers should be avoided [16, 53]. See Table 6 for details about the classification of coadministered drugs and precautions for everolimus.

**Table 6:** Precautions for coadministration of everolimus with other drugs [16]

Classification	Drug names	Precautions for everolimus
Strong CYP3A4 and/or PgP inducers^a^	Including but not limited to ketoconazole, itraconazole, clarithromycin, atazanavir, nefazodone, saquinavir, telithromycin, ritonavir, indinavir, nelfinavir, and voriconazole.	Strong CYP3A4 inhibitors increase the plasma concentration of everolimus. Coadministration should be avoided.
Moderate CYP3A4 and/or PgP inhibitors^a^	Including but not limited to amprenavir, fosamprenavir, aprepitant, erythromycin, fluconazole, verapamil, and diltiazem.	Moderate CYP3A4 inhibitor increases the plasma concentration of everolimus. These drugs should be coadministered with caution. For those who must coadminister moderate CYP3A4 and/or PgP inhibitors, the dose of everolimus should be decreased to 2.5 mg/day. If well tolerated, consider increasing the dose from 2.5 mg/day to 5 mg/day. When discontinuing the moderate CYP3A4 and/or PgP inhibitors, a wash‐out period of 2‐3 days should be allowed before increasing the dose of everolimus. After the discontinuation, the dose of everolimus should be increased to the same dose before the moderate CYP3A4 and/or PgP inhibitors were added.
Strong CYP3A4 inducers^b^	Including but not limited to phenytoin, carbamazepine, rifampin, rifabutin, rifapentine, and phenobarbital.	Strong CYP3A4 inducers decrease the plasma concentration of everolimus. Coadministration should be avoided. When strong CYP3A4 inducers must be coadministered, consider doubling the daily dose of everolimus using increments of 5 mg or less. If the strong inducer is discontinued, consider a washout period of 3 to 5 days, before the everolimus dose is returned to the dose used before initiation of the strong CYP3A4/PgP inducer

Abbreviation: PgP, P‐glycoprotein.

^a^ When on everolimus, foods with known cytochrome P450 and PgP inhibiting effects should be avoided, such as grapefruit, grapefruit juice, and so forth.

^b^ St. John's wort (*Hypericum perforatum*) can decrease the exposing dose of everolimus unexpectedly, which should also be avoided.

### 3.5 Use in specific populations

#### 3.5.1 PI3K inhibitors (alpelisib)

Based on the findings in animal experiments and studies on its mechanisms of action, alpelisib can cause fetal harm when administered to pregnant women, so pregnant patients should be informed of the potential risks to the fetus. Serious adverse effects can also be brought onto the breastfeeding infants, so for lactating women, breastfeeding should be avoided during alpelisib treatment and a week after the last dose [14]. For women of childbearing age, effective contraceptive measures should be taken during alpelisib treatment and a week after the last dose [14]. For the elderly population (≥65 years old), the incidence of Grades 3 and 4 hyperglycemia is higher than in those younger than 65 years old (44% vs. 32%), so close monitoring for adverse effects is recommended and dosage should be modified accordingly [14]. As for patients with mild to moderate renal impairment, no dose modification is required, while for those with severe renal impairment, no empirical data are available, and alpelisib should be administered with caution. For patients with liver impairment, no dose modification is needed [14]. After taking the adverse effects of alpelisib into consideration, its use in specific populations is presented in Table 7.

**Table 7:** Contraindications of alpelisib in specific populations [14]

Drug	Pregnant women	Lactating women	Women of childbearing age	Elderly patients (≥65 years old)	Renal impairment	Liver impairment	Allergy
Alpelisib	Prohibited	Use with caution	Use with caution	Can be used	Can be used in mild to moderate renal impairment, but should be used with caution in severe renal impairment	Can be used	Prohibited if allergic to alpelisib or any of the components in the tablet

#### 3.5.2 mTOR inhibitors (everolimus)

For pregnant patients, the embryotoxicity of everolimus has been found in mechanism studies. Thus, everolimus should not be used in pregnant patients unless its benefits outweigh the potential risks to the fetus. Serious adverse effects can also be brought onto the breastfeeding infants, so for lactating women, breastfeeding should be avoided during everolimus treatment and 2 weeks after the last dose [16]. For women of childbearing age, effective contraceptive measures should be taken during everolimus treatment and 8 weeks after the last dose [16]. When used in the elderly population (≥65 years old), the incidence of adverse effects that lead to drug discontinuation is higher than in the younger population (33% vs. 17%), so close monitoring for adverse effects is recommended and dosage should be modified accordingly [16]. For patients with mild (Child‐Pugh class A) or moderate (Child‐Pugh class B) liver impairment, a decreased dose is recommended [16]. After taking the adverse effects of everolimus into consideration, its use in specific populations is presented in Table 8.

**Table 8:** Contraindications of everolimus in specific populations [16]

Drug	Pregnant women	Lactating women	Women of childbearing age	Elderly patients (≥65 years old)	Renal impairment	Liver impairment	Allergy
Everolimus	Prohibited	Use with caution	Use with caution	Use with caution	Can be sued	Use with caution^a^	Prohibited if allergic to everolimus or any of the components in the tablet

^a^ Liver impairment can increase the exposure to everolimus. Thus, recommended dose modification is listed as follows. (1) Mild liver impairment (Child‐Pugh class A): recommended dose is 7.5 mg/day; if not well‐tolerated, consider lowering the dose to 5 mg/day. (2) Moderate liver impairment (Child‐Pugh class B): recommended dose is 5 mg/day; if not well‐tolerated, consider lowering the dose to 2.5 mg/day. (3) Severe liver impairment (Child‐Pugh class C): if the expected benefit outweighs the risks, everolimus can be administered at 2.5 mg/day, but the dose should not exceed this level. Over the course of everolimus treatment, if there is any change in the liver function status (Child‐Pugh score), the dose should be modified accordingly.

### 3.6 Monitoring parameters and timing before/during drug administration

#### 3.6.1 PI3K inhibitors (alpelisib)

Monitoring parameters, frequencies, and related recommendations on clinical management are summarized in Table 9, which is in accordance with the FDA drug label [14]. Fasting blood glucose and HbA1c should be monitored before and after alpelisib treatment.

**Table 9:** Recommendations on monitoring parameters and frequencies in patients receiving alpelisib [14]

Parameters	Alpelisib
FPG^a^	Blood glucose should be monitored before the initiation of alpelisib treatment.
	After initiation, blood glucose and/or FPG should be monitored at least once a week in the first 2 weeks, and once every four weeks after that. Additional tests are also recommended if considered necessary.
HbA1c	In the beginning, HbA1c should be monitored before the initiation of alpelisib treatment.
	Over the course of alpelisib treatment, HbA1c should be monitored at least once every 3 months. Additional tests are also recommended if considered necessary.

Abbreviation: HbA1c, hemoglobin A1c, glycated hemoglobin.

^a^ FPG, fasting plasma glucose. Blood glucose status should be monitored over the course of alpelisib treatment. If the blood glucose level is examined after the initiation of alpelisib treatment, instead of FPG, it should be fasting blood glucose.

#### 3.6.2 mTOR inhibitors (everolimus)

Monitoring parameters, frequencies, and related recommendations on clinical management are summarized in Table 10, which is in accordance with the FDA drug label [16]. Renal function, blood glucose, lipid panel, and hematologic parameters should be monitored before and after everolimus treatment.

**Table 10:** Recommendations on monitoring parameters and frequencies in patients receiving everolimus [16]

Parameters	Everolimus
Renal function	Elevated blood creatinine levels and proteinuria have been reported in patients receiving everolimus. Monitoring of renal function before the initiation of everolimus treatment is recommended, including blood urea nitrogen (BUN), urine protein, or blood creatinine tests, as well as regular tests afterward. For those with risk factors for further renal impairment, special attention should be paid to their renal function.
Blood glucose and lipid panel	For patients who already have hyperglycemia, hypercholesterolemia, and hypertriglyceridemia, fasting blood glucose and lipid panel should be examined before the initiation of everolimus treatment. Regular tests are also recommended after initiation, and appropriate management should be provided. If everolimus is coadministered with other medications that might cause hyperglycemia, more frequent tests are recommended. If possible, ideal blood glucose control should be obtained before initiation.
Hematologic parameters	Decreased hemoglobin, lymphocytes, and platelets have been reported in patients receiving everolimus. It is recommended that a complete blood count should be examined before the initiation of everolimus treatment, and it should also be tested regularly afterward.

### 3.7 Management of adverse reactions

#### 3.7.1 PI3K inhibitors (alpelisib)

Most common adverse reactions (of all grades, with an incidence of ≥20%) of alpelisib in patients with PIK3CA‐mutated, HR‐positive/HER2‐negative advanced or metastatic breast cancer include elevated blood glucose, elevated creatinine, diarrhea, skin rash, decreased lymphocyte count, and so forth. Most common Grades 3 or 4 adverse reactions (with an incidence of ≥2%) include hyperglycemia, skin rash, diarrhea, and so forth [14]. The management of alpelisib‐associated adverse reactions over the course of treatment should be consistent with the grading [14], with details in Table 11.

**Table 11:** Recommendations on dose modification and management of adverse reactions during alpelisib therapy [14, 59]

Adverse reactions	Severity^a^	Recommendations on dose modification and management
Hyperglycemia	Grade 1 (ULN < FPG ≤ 160 mg/dl [8.9 mmol/L])	No need to modify the dose of alpelisib. Make dietary adjustments (low‐carbohydrate diet) and increase physical activity first. Initiate metaformin treatment.
	Grade 2 (160 < FPG ≤ 250 mg/dl [8.9–13.9 mmol/L])	No need to modify the dose of alpelisib. Make dietary adjustments (low‐carbohydrate diet) and increase physical activity first. Initiate or intensify metformin treatment. Add SGLT‐2 inhibitors or other anti‐hyperglycemic agents.
	Grade 3 (250 < FPG ≤ 500 mg/dl [13.9–27.8 mmol/L])	If FPG cannot be lowered to ≤160 mg/dL or 8.9 mmol/L within 21 days with appropriate anti‐hyperglycemic treatment, the dose of alpelisib should be lowered. Refer to the recommendations below for exact insulin dosage based on abnormal FPG values.
	Grade 4 (FPG > 500 mg/dl [27.8 mmol/L])	Discontinue alpelisib. Make dietary adjustments (low‐carbohydrate diet) and increase physical activity first. Initiate or intensify metformin treatment. Add or resume SGLT‐2 inhibitors or other anti‐hyperglycemic agents until hyperglycemia improves. Provide intravenous rehydration and consider other appropriate management (such as interventions for electrolyte imbalances, ketoacidosis, or hyperglycemic hyperosmolar state).
		If FPG cannot be lowered to ≤160 mg/dL or 8.9 mmol/L within 3‐5 days with appropriate anti‐hyperglycemic treatment, the dose of alpelisib should be lowered. Then, if FPG cannot be lowered to ≤160 mg/dL or 8.9 mmol/L within 3‐5 days with appropriate anti‐hyperglycemic treatment, consultation with experienced endocrinologists should be arranged. Then, if FPG cannot be lowered to ≤160 mg/dL or 8.9 mmol/L within 21 days with appropriate anti‐hyperglycemic treatment, permanently discontinue alpelisib.
		Discontinue alpelisib. Initiate or intensify appropriate anti‐hyperglycemic treatment. Provide intravenous rehydration and consider other appropriate management (such as interventions for electrolyte imbalances, ketoacidosis, or hyperglycemic hyperosmolar state). Repeat FPG test within 24 h, if necessary.
		If FPG is lowered to ≤500 mg/dL or 27.8 mmol/L, follow the recommendations for grade 3 hyperglycemia. If FPG is stays > 500 mg/dL or 27.8 mmol/L, permanently discontinue alpelisib.
Skin rash	Grade 1 (Skin reaction involving <10% of body surface area [BSA])	No need to modify the dose of alpelisib. Initiate topical steroid therapy. Consider additional oral anti‐histamines for symptom control.
	Grade 2 (Skin reaction involving 10%–30% of BSA)	No need to modify the dose of alpelisib. Initiate or intensify topical steroid and oral anti‐histaminic therapies. Consider systemic corticosteroid therapy.
	Grade 3 (Skin reaction involving >30% of BSA, e.g., severe skin rash unresponsive to medications)	Discontinue alpelisib. Initiate or intensify topical/systemic corticosteroid and oral anti‐histaminic therapies. Once the skin reaction is alleviated to grade 1 or lower, reinstitute alpelisib at the same dose as when the rash first appears, and then lower the dose when the rash appears again.
	Grade 4 (Severe generalized infection requiring intravenous antibiotics or skin reactions with life‐threatening outcomes, involving any percentage of BSA, e.g., bullous, vesicular, or exfoliative skin diseases)	Permanently discontinue alpelisib.
Diarrhea	Grade 1	No need to modify the dose of alpelisib. Initiate appropriate medication and monitoring if necessary.
	Grade 2	Initiate or intensify medication and monitoring as appropriate if necessary. Discontinue alpelisib until the diarrhea is alleviated to grade 1 or lower. Then, reinstitute alpelisib at the same dose.
	Grade 3 and 4	Initiate or intensify medication and monitoring as appropriate if necessary. Discontinue alpelisib until the diarrhea is alleviated to grade 1 or lower. Then, reinstitute alpelisib at a lower dose.
Noninfectious pneumonitis	New‐onset or worsening respiratory symptoms, or suspicion of existing noninfectious pneumonitis	Discontinue alpelisib immediately and perform assessments for noninfectious pneumonitis.
	Confirmed noninfectious pneumonitis	Permanently discontinue Alpelisib.
Other toxicities	Grades 1 or 2	No need to modify the dose of alpelisib. Initiate appropriate medication and monitoring if necessary.
	Grade 3	Discontinue alpelisib until the reaction is alleviated to grade 1 or lower. Then, reinstitute alpelisib at a lower dose.
	Grade 4	Permanently discontinue Alpelisib.
Rapid‐acting insulins (aspart or lispro)		No (weight gain)
	300–440 mg/dl	450–599 mg/dl
eGFR ≥40	0.20 units/kg × 1 dose	0.25 units/kg × 1 dose
eGFR <40	0.10 units/kg × 1 dose	0.15 units/kg × 1 dose

Abbreviation: eGFR, estimated glomerular filtration rate.

^a^ Grading of adverse events, Grade 1 = mild symptoms, Grade 2 = moderate symptoms, Grade 3 = severe symptoms, Grade 4 = life‐threatening symptoms.

##### Hyperglycemia

The incidence of alpelisib‐associated hyperglycemia is 65%, and the incidences of Grades 3 and 4 hyperglycemia are 33% and 3.9%, respectively [14], with a higher incidence during the first 1–2 months of alpelisib administration, which can be managed with anti‐hyperglycemic agents. When the dose of alpelisib is as low as 200 mg, the incidence of hyperglycemia is greatly reduced. This hyperglycemia is also reversible after discontinuation of alpelisib [14]. For patients with a history of diabetes, blood glucose should be closely monitored and anti‐hyperglycemic treatment should be intensified [14]. Alpelisib can be an option for patients with type 2 diabetes and good blood glucose control, while its safety profile is still unclear in patients with type 1 diabetes or patients with type 2 diabetes and suboptimal blood glucose control [14]. The PI3K pathway activates AKT and downstream pathways, participating in sugar and lipid metabolism and inhibiting gluconeogenesis, which explains the mechanism of alpelisib‐induced hyperglycemia [54, 55]. Preclinical data have shown that inhibition of PI3Kα stimulates liver glycogenolysis and decreases signal transduction of insulin receptors, resulting in insulin resistance and hyperglycemia [56, 57, 58].

Expert panel recommendations: Before the initiation of alpelisib treatment, FPG and HbA1c should be tested, and blood glucose control should be optimized in advance if necessary. After initiation, regular monitoring is recommended, and initiation or optimization of anti‐hyperglycemic treatment should be considered according to the clinical parameters (Tables 9 and 11). Currently, metformin is the drug of choice for alpelisib‐associated hyperglycemia, as it somewhat increases the sensitivity to insulin. Moreover, due to the desensitization of insulin caused by alpelisib, there is usually an increase in the dose of insulin. There is also evidence supporting the effectiveness of SGLT‐2 inhibitors, thiazolidinediones, and glucagon‐like peptide‐1 (GLP‐1) analogs in controlling alpelisib‐associated hyperglycemia. If a patient has suboptimal blood glucose control, endocrinology consultation should be arranged as soon as possible, to obtain a plan for blood glucose management.

##### Skin rash

Among patients who experienced Grades 2 or 3 skin rash, the median time to the first onset is 12 days [14]. In the SOLAR‐1 trial, preventive medication can lower the incidence and mitigate the severity of skin rash, compared to those who did not receive anti‐rash medication (rash of any grade: 26.7% vs. 64.1%; rash of Grade 3: 11.6% vs. 22.7%) [14]. Therefore, preventive management has a positive impact on modifying the development and severity of skin rash.

Expert panel recommendations: Preventive anti‐histaminic medication before the initiation of alpelisib is recommended, such as cetirizine (10 mg q.d.), loratadine (10 mg q.d.), and so forth, to lower the risk and severity of skin rash. Patients should be advised to use gentle, unscented soap and laundry detergent, as well as noncomedogenic skin moisturizer and sunscreen, to prevent dry skin. Unprotected sun exposure and skincare products containing irritative components, such as alcohol, salicylic acid, ammonium lactate, and urea should also be avoided. Most of the rashes are reversible after appropriate treatment and discontinuation of alpelisib. Symptomatic treatment and severity‐appropriate management should be undertaken according to the type and severity of skin rash (Tables 11 and 12). Anti‐infective medications can also be administered if the infection is present. For the skin rash that cannot be relieved with conventional treatment, a dermatology consultation should be arranged in time, to obtain a plan of management.

**Table 12:** Common anti‐rash regimens [59]

Class	Drug	Route of administration	Recommended dose	Indications
Sunscreen	Broad‐spectrum mineral sunscreen	Topical	SPF ≥ 15	Face or other exposed areas
Nonsedating anti‐histamines	Cetirizine	Oral	10 mg once daily	Prevention
			10 mg twice daily	Rash of Grade 1 or higher
	Loratadine	Oral	10 mg once daily	Prevention
			10 mg twice daily	Rash of Grade 1 or higher
	Fexofenadine	Oral	180 mg once daily	Prevention
			180 mg twice daily	Rash of Grade 1 or higher
Sedating anti‐histamines	Diphenhydramine	Oral	25–50 mg before bedtime	For burning, tingling, or pruritus
	Hydroxyzine	Oral	25 mg before bedtime	
Corticosteroids	Triamcinolone ointment or cream	Topical	0.1%	Grades 1 or 2 rash
	Fluocinolone acetonide ointment or cream	Topical	0.05%	Grades 1 or 2 rash
	Prednisone	Oral	0.5–1 mg/kg/d or equivalent doses	Grade 3 rash
GABA agonists	Gabapentin	Oral	300 mg three times daily	
	Pregabalin	Oral	50 mg twice daily	

##### Diarrhea

The incidence of alpelisib‐associated diarrhea is 58%, and the median time to onset of Grades 2 or 3 diarrhea is 46 days. Severe diarrhea might occur in patients on alpelisib, resulting in dehydration and acute kidney injury [14]. For diarrhea of different levels of severity, management should be provided according to Table 11, such as skipping a dose, lowering the dose, or discontinuation of alpelisib. If diarrhea occurs during alpelisib treatment, patients should be advised to start antidiarrheal treatment and oral rehydration, and their healthcare decision‐makers should also be informed [14].

##### Noninfectious pneumonitis

Severe noninfectious pneumonitis has been reported in patients receiving alpelisib treatment, including acute interstitial pneumonitis and interstitial lung disease [14]. About noninfectious pneumonitis was found in 1.8% of the patients receiving alpelisib [14]. Thus, for patients with nonspecific respiratory signs and symptoms (such as hypoxia, cough, dyspnea, and interstitial lung infiltration on radiologic examinations, after excluding infection, tumor, and other etiologies), noninfectious pneumonitis should be considered [14].

##### Other adverse reactions

Other adverse reactions, such as nausea (45%), fatigue (42%), loss of appetite (36%), stomatitis (30%), and vomiting (27%) have also been reported in patients receiving alpelisib [14]. In general, for adverse reactions of Grades 1 or 2, no dose modification is needed, but medication and monitoring can be undertaken if necessary. For adverse reactions of Grade 3, alpelisib should be discontinued until the reactions are alleviated to Grade 1 or lower. Then, alpelisib should be reinstituted at a lower dose. However, when adverse reactions of Grade 4 occur, alpelisib should be permanently discontinued [14].

##### Severe hypersensitivity

The incidence of alpelisib‐associated Grades 3 or 4 hypersensitivity is 0.7% [14]. Patients should be informed of the signs and symptoms of severe hypersensitivity (including but not limited to dyspnea, flushing, skin rash, fever, and tachycardia). Alpelisib should be permanently discontinued when severe hypersensitivity occurs [14].

##### Severe skin reaction

Stevens‐Johnson syndrome (SJS) and erythema multiforme (EM) (both are severe skin reactions) have been reported in about 0.4% and 1.1% of patients receiving alpelisib, respectively [14]. Thus, alpelisib treatment should not be instituted in patients with a history of SJS, EM, or toxic epidermal necrolysis (TEN). Patients should be informed of the signs and symptoms of severe skin reactions (such as prodromal fever, flu‐like symptoms, mucosal lesions, or progressively worsening skin rash) [14]. When signs or symptoms of severe skin reactions occur, alpelisib should be discontinued until the etiology of the skin reaction is determined and dermatology consultation is also advisable. If SJS, TEN, or EM is confirmed, alpelisib should be permanently discontinued. For patients who have ever experienced severe skin reactions during alpelisib treatment, alpelisib should never be reinstituted. If the skin reactions are not severe enough to be diagnosed as SJS, TEN, or EM, the dose of alpelisib may be modified and topical corticosteroids or oral anti‐histamines are indicated [14].

#### 3.7.2 mTOR inhibitors (everolimus)

Most common adverse reactions (of all grades, with an incidence of ≥30%) of everolimus in patients with HR‐positive/HER2‐negative advanced breast cancer include stomatitis, infection, skin rash, fatigue, diarrhea, and loss of appetite. Most common Grades 3 or 4 adverse reactions (with an incidence of ≥2%) include stomatitis, infection, hyperglycemia, fatigue, dyspnea, pneumonia, and diarrhea [16, 18, 19, 20, 21, 48, 49, 60]. Most of the everolimus‐associated adverse reactions are of Grades 1 or 2, occur at the early stage of everolimus therapy, and can be controlled by decreasing the dose or by discontinuing everolimus [18, 19, 20, 21, 48, 49, 60, 61, 62]. Good management of the adverse reactions can improve compliance and thus maintain its effectiveness. The management of alpelisib‐associated adverse reactions over the course of treatment should be consistent with the grading, as shown in Table 13.

**Table 13:** Recommendations on dose modification and management of adverse reactions during everolimus therapy [16, 69]

Adverse reactions	Severity^a^	Recommendations on dose modification^b^ and management
Stomatitis [16]	Grade 1 (Asymptomatic or mildly symptomatic, no intervention required)	No need for dose modification. Gargle with alcohol‐free water or normal saline (0.9%) several times a day.
	Grade 2 (Moderate pain, swallowing unaffected, texture‐modified food required)	Temporarily discontinue everolimus until the symptoms are alleviated to Grade 1 or lower. Reinstitute everolimus at the same dose.
		If Grade 2 stomatitis recurs, discontinue everolimus until symptoms are alleviated to Grade 1 or lower. Reinstitute everolimus at a lower dose. Treat with topical analgesics (such as benzocaine, butamben, tetracaine hydrochloride, menthol, or phenol). Add topical corticosteroids as appropriate (such as triamcinolone oral patch)^c^.
	Grade 3 (Severe pain, swallowing affected)	Temporarily discontinue everolimus until the symptoms are alleviated to Grade 1 or lower. Reinstitute at a lower dose. Treat with topical analgesics (such as benzocaine, butamben, tetracaine hydrochloride, menthol, or phenol). Add topical corticosteroids as appropriate (such as triamcinolone oral patch)^c^.
	Grade 4 (With life‐threatening outcomes, emergent intervention is required)	Terminate everolimus therapy. Provide appropriate treatment.
Noninfectious pneumonitis [16, 24011786]	Grade 1 (Asymptomatic, found in clinical examinations [CT], no intervention required)	No need for dose modification. Exclude infectious etiologies and monitor closely.
	Grade 2 (Symptomatic, medication required, activities of daily life unaffected)	Consider discontinuation. Obtain chest CT, exclude infectious etiologies, and consider corticosteroid treatment until symptoms are alleviated to Grade 1 or lower. Reinstitute everolimus at a lower dose.
		Terminate everolimus therapy if no relief is observed after 4 weeks.
	Grade 3 (Severe symptoms, daily life affected, oxygen supplementation required)	Discontinue everolimus. Obtain chest CT, exclude infectious etiologies, and consider corticosteroid treatment until symptoms are alleviated to grade 1 or lower. Reinstitute everolimus at a lower dose.
		Obtain pulmonary function test and bronchoscopic examination with bronchoalveolar lavage and/or biopsy.
		If grade 3 toxicities recur after reinstitution, consider terminating everolimus therapy.
	Grade 4 (Life‐threatening impairment of respiratory function, emergent intervention required [such as tracheostomy or intubation])	Terminate everolimus therapy. Obtain chest CT, exclude infectious etiologies, and treat with corticosteroids. Obtain pulmonary function test and bronchoscopic examination with bronchoalveolar lavage and/or biopsy.
Other nonhematologic toxicities (Except metabolic events) [16]	Grade 1	If tolerated, no need for dose modification. Treat and monitor as appropriate.
	Grade 2	If tolerated, no need for dose modification. Treat and monitor as appropriate.
		If not tolerated, temporarily discontinue everolimus until symptoms are alleviated to Grade 1 or lower. Reinstitute everolimus at the same dose.
		If Grade 2 event recurs, discontinue everolimus until symptoms are alleviated to Grade 1 or lower. Reinstitute everolimus at a lower dose.
	Grade 3	Temporarily discontinue everolimus until symptoms are alleviated to Grade 1 or lower. Treat and monitor as appropriate. Consider reinstitution at a lower dose.
		If Grade 2 event recurs, consider termination of everolimus therapy.
	Grade 4	Terminate everolimus therapy. Treat as appropriate.
Metabolic events (e.g., hyperglycemia and dyslipidemia) [16, 65, 68]	Grade 1 (e.g., for hyperglycemia, fasting blood glucose between ULN and 160 mg/dl, and for hypercholesterolemia, cholesteral between ULN and 2.5× ULN)	No need for dose modification. Treat and monitor as appropriate.
	Grade 2 (e.g., for hyperglycemia, fasting blood glucose is between 160 and 250 mg/dl, and for hypercholesterolemia, cholesterol is between 2.5× ULN and 5× ULN)	No need for dose modification. Treat and monitor as appropriate.
	Grade 3 (e.g., for hyperglycemia, fasting blood glucose is between 250 and 500 mg/dl, and for hypercholesterolemia, cholesterol is between 5× ULN and 10× ULN)	Temporarily discontinue everolimus and reinstitute it at a lower level. Treat and monitor as appropriate.
	Grade 4 (e.g., for hyperglycemia, fasting blood glucose >500 mg/dl, and for hypercholesterolemia, cholesterol >10× ULN)	Terminate everolimus therapy. Treat as appropriate.

Abbreviation: ULN, upper limit of normal.

^a^ Grading of adverse events, Grade 1 = mild symptoms, Grade 2 = moderate symptoms, Grade 3 = severe symptoms, Grade 4 = life‐threatening symptoms.

^b^ If a lower dose is needed, it is recommended to administer everolimus at about 50% of the previous dose. If the daily dose is smaller than a tablet, consider administering everolimus every other day.

^c^ When treating stomatitis, avoid using products containing hydrogen peroxide, iodine, and thyme derivatives because these may worsen oral ulcers.

##### Stomatitis

Special attention should be paid to oral mucositis during everolimus therapy, which usually manifests as clearly demarcated, gray, elliptical, cold sore‐like ulcers surrounded by a red halo [63]. Due to its destructive effects on the mucosal barrier, the risk of systemic infection is increased. Clinical data shows that the incidence of everolimus‐associated stomatitis is 33.8%–69% and the incidence of Grades 3 or 4 stomatitis is 2.9%–11% [16, 18, 19, 20, 21, 48, 49, 60]. Stomatitis is associated with the immunosuppressive effects of everolimus, and its incidence and severity can be reduced by gargling with alcohol‐free water or saline water and applying topical analgesics or steroids so that dose reduction or even discontinuation can be avoided [16]. The SWISH trial, a Phase II clinical trial [64] confirms that in a postmenopausal patient with HR‐positive/HER2‐negative advanced breast cancer receiving everolimus plus endocrine therapy, preventive use of glucocorticoid‐containing mouthwash could decrease the risk of stomatitis. Compared to the BOLERO‐2 trial, the incidences of stomatitis in all grades (21.2% vs. 67%) and of Grade 2 or higher (2.4% vs. 33%) are both remarkably decreased at Week 8.

Expert panel recommendations: For mild stomatitis, topical supportive treatment is recommended in general, including gargling with alcohol‐free water or saline water and a cold compress. For moderate to severe stomatitis, topical medications may be added, such as local anesthetics (e.g., lidocaine), glucocorticoids, growth factors, Kangfuxin solution, and so forth. Meanwhile, when deemed appropriate, systemic medications (antibiotics, antifungals, antivirals, analgesics, etc.) can also be added. If necessary, everolimus therapy should be discontinued or terminated (Table 13). Prevention of stomatitis is just as important as treatment, which requires gargling with steroid‐containing mouthwash before everolimus therapy, as well as complete dental examination, regular oral care and cleansing, healthy oral hygiene habits, and avoidance of hot, acidic, spicy, hard, crispy, or other irritative food during everolimus therapy.

##### Infection

The risk of infection is increased by the immunosuppressive effects of everolimus, and the possible infectious agents include bacteria, fungi, viruses, and parasites, including opportunistic infections [16]. Both local and systemic infections have been reported in patients receiving everolimus, including pneumonia, mycobacterial infections, other bacterial infections, invasive fungal infections (such as aspergillosis, candidiasis, and *Pneumocystis jiroveci* pneumonia [PJP]), and viral infections (such as reactivation of hepatitis B virus). Some of the infections are severe or life‐threatening (such as those leading to sepsis, respiratory failure, or liver failure) [16]. Before the initiation of everolimus therapy, the pre‐existing invasive fungal infections should be thoroughly treated, and signs of symptoms of infection should be monitored during everolimus therapy. If diagnosed as infection, appropriate treatment should be initiated rapidly and discontinuation or termination of everolimus therapy should be considered [16]. Cases of PJP have been reported in patients receiving everolimus, with death being the outcome in some of them. Concomitant administration of corticosteroids or other immunosuppressants might be associated with PJP. Thus, preventive treatment for PJP should be considered when concomitant corticosteroids or other immunosuppressants are required [16].

##### Skin rash

Skin rash is a common everolimus‐associated cutaneous toxicity, with an incidence of 27%–44%, and the incidence of Grades 3 or 4 skin rash is less than 1%–4%, which usually occurs in the early stage of everolimus therapy [16, 18, 19, 20, 21, 48, 49, 60]. Most everolimus‐associated skin rash resolves without intervention [65]. Symptoms can also be relieved by topical application of hydrocortisone ointment or moisturizer. However, for patients susceptible to infection and/or hyperglycemic patients, steroids should be used with caution in the treatment of skin rash [65].

##### Noninfectious pneumonitis

pneumonitis is a common adverse reaction associated with everolimus. According to the international trials of BOLERO‐2 [60] and BOLERO‐4 [20], the incidences of everolimus‐associated pneumonitis are 16% and 18%, respectively, while in the BOLERO‐5 trial [19] conducted in China, the incidence of everolimus‐associated noninfectious pneumonitis is 23.8%, slightly higher than the aforementioned international trials. Nonetheless, everolimus‐associated lung toxicity is not lethal and is correlated with its efficacy. The YOUNGBC‐5 trial [61], a smaller‐scale, single‐center, retrospective study in China suggests that everolimus‐associated pneumonitis was found in over half of the patients with advanced breast cancer receiving everolimus therapy, more than 80% of which occurred during the early stage of everolimus therapy. However, only less than one‐third of the patients with everolimus‐associated pneumonitis shown on radiologic images were actually symptomatic, and the symptoms resolved after discontinuation of everolimus in most of these patients. Compared to patients who did not experience everolimus‐associated pneumonitis, those who did benefited more from everolimus in terms of survival.

Expert panel recommendations: The following precautions should be taken before the initiation of everolimus therapy [66]. (1) Obtain a thorough history of respiratory diseases. (2) Obtain a recent chest X‐ray image or baseline high‐resolution CT scan (if available). (3) Instruct the patient to report any new or worsening respiratory symptoms (such as cough, tachypnea, and fever) in a timely manner. (4) Advise the patient to take a pulmonary function test if there are respiratory symptoms. (5) Use everolimus with caution and follow up closely if there is a pre‐existing diffuse interstitial lung disease. Pathologic diagnosis of interstitial pneumonitis should only be made with a multidisciplinary approach after taking clinical symptoms, imaging evidence, and pathologic findings into consideration. High‐resolution CT provides reliable evidence for clinical diagnosis and differential diagnosis. Multidisciplinary consultations are recommended, if necessary [67].

If asymptomatic noninfectious pneumonitis is found but there are changes in the disease course, additional attention should be paid. If there is new‐onset, symptomatic noninfectious pneumonitis, it is recommended that corticosteroid be used until the symptoms are alleviated to Grade 1 or lower with close monitoring. Everolimus should be discontinued temporarily if there is severe interstitial lung disease or pneumonitis. Reinstitution from a lower dose can be considered after the symptoms resolve. For adverse reactions not responsive to conventional treatment, timely consultation with specialists and multidisciplinary management is recommended.

##### Hyperlipidemia

The incidences of everolimus‐associated hypertriglyceridemia and hypercholesterolemia are 17%–57.4% and 23.8%–51.5%, respectively, and the incidences of Grades 3 or 4 hypertriglyceridemia and hypercholesterolemia are 2%–12.9% and 0%–1.0%, respectively [18, 19, 20, 21]. Lipid panel should be examined before receiving everolimus therapy (fasting serum cholesterol should be ≤300 mg/dl and fasting triglyceride should be ≤2.5× ULN). Patients with baseline dyslipidemias should receive lipid‐lowering treatment according to the guidelines, to meet these criteria [65, 68]. Hypertriglyceridemia of ≥500 mg/dl indicates an elevated risk for acute pancreatitis, and immediate treatment with fibrates is necessary [65, 68].

##### Hyperglycemia

The incidence of everolimus‐associated hyperglycemia is 14%–42.5% and the incidence of Grades 3 or 4 hyperglycemia is 3%–10% [16, 17, 18, 19, 20, 21, 49, 60]. For those with carbohydrate metabolism disorders, fasting blood glucose levels should be monitored before receiving everolimus treatment and blood glucose control should be optimized according to the standardized management in the guidelines [65]. The glucose‐lowering medication of choice is metformin and other options include SGLT‐2 inhibitors (dapagliflozin, empagliflozin, etc.), pioglitazone, and so forth. Insulin should be added in time if ideal blood glucose control cannot be achieved [68]. An endocrinology consultation should be arranged if necessary.

#### 3.7.3 General principles of managing adverse reactions

Management of adverse reactions is essential to a patient's quality of life. As each targeted agent usually has a distinctive spectrum of adverse reactions, a comprehensive understanding of the potential adverse reactions for different PAM pathway inhibitors is important and close attention should be paid. Early prevention, close monitoring, and timely, appropriate, and effective interventions should be implemented. As for severe adverse reactions, PAM pathway inhibitors should be temporarily or permanently discontinued. Another critical aspect of managing adverse reactions involves multidisciplinary teams. The collaboration between endocrinologists, dermatologists, gastroenterologists, and cardiologists should be strengthened, which is of great significance in the management of adverse reactions during treatment with PAM pathway inhibitors.

### 3.8 Standards for PIK3CA mutation testing

#### 3.8.1 Overview of the PIK3CA gene

As the initiating molecule of the PAM pathway, PI3K molecules can be categorized into Classes I, II, and III, of which Class I PI3K is the driving factor for cancer. Class I PI3K can be further divided into Class IA and Class IB [24]. Of these two classes, Class IA is a heterodimer formed by a catalytic subunit p110 and the regulatory subunit p85 [26]. For PI3K Class IA, the isozymes p110α, p110β, and p110δ are encoded by PIK3CA, PIK3CB, and PIK3CD, respectively [26]. In contrast, PI3K class IB is formed by p110γ only, encoded by PIK3CG. The PIK3CA gene encodes p110α protein, one of the catalytic subunits of the PI3K enzyme, playing a vital role in the pathogenesis of cancer driven by PIK3CA mutations, as well as in the development of resistance in breast cancer [70, 71, 72]. Most of the PIK3CA mutations occur in the “hot‐spots” of the gene sequence, including E542K ad E545K in exon 9 (helical domain) and H1047R in exon 20 (kinase domain) [70, 73]. Multiple clinical studies revealed that the prevalence of PIK3CA mutations is 18%–46.5% in breast cancer [15, 74, 75, 76, 77, 78, 79, 80, 81]. It is also reported by clinical studies on mutation analysis in patients with breast cancer in China that PIK3CA is also common in Chinese patients with breast cancer, with a prevalence of 32%–46.5% [15, 80, 81]. According to a clinical study published on *Breast Cancer* in 2021, compared to the TCGA datasets, which mainly consist of data from patients with breast cancer in western countries, the prevalence of PIK3CA mutation in patients with breast cancer in China is higher (45.6% vs. 34.7%, *p* < 0.001). Among the three common mutation loci (p.E545K, p.E542K, and p.H1047R), p.H1047R is the most common one (66.1%). Among the four molecular subtypes of breast cancer, ER‐positive/HER2‐positive breast cancer has the highest PIK3CA mutation rate (51.6%), followed by ER‐positive/HER2‐negative breast cancer (48.7%), and ER‐negative/HER2‐negative breast cancer has the lowest mutation rate (30.0%) but the highest PIK3CA amplification rate (19.05%) [15].

#### 3.8.2 Indicated population of PIK3CA mutation testing and types of specimens

According to *NCCN Clinical Practical Guidelines in Oncology. Breast Cancer. Version 1.2022*, *Endocrine Treatment and Targeted Therapy for Hormone Receptor‐Positive, Human Epidermal Growth Factor Receptor 2‐Negative Metastatic Breast Cancer: ASCO Guideline Update*, and 5*th ESO‐ESMO International Consensus Guidelines for Advanced Breast Cancer (ABC 5)*, for patients with HR‐positive/HER2‐negative advanced or metastatic breast cancer, tissue biopsy for PIK3CA mutation testing is indicated when there is disease progression after endocrine‐based therapy [38, 82, 83].

The PIK3CA mutation testing can be performed with either cancer tissue or plasma ctDNA specimens. Tissue biopsy specimens are preferred for patients with advanced breast cancer. If PIK3CA is not detected in the plasma ctDNA specimen, it is recommended to obtain cancer tissue specimens to clarify the PIK3CA mutation status [38, 82, 83, 84]. Due to the similar distribution of PIK3CA mutation in primary and metastatic sites, either fresh biopsy tissue samples or archived tissue samples can be used for mutation testing [85]. As reported in the SOLAR‐1 trial, whether PIK3CA mutation is detected in plasma ctDNA or in cancer tissue, patients can benefit remarkably from alpelisib and fulvestrant combination therapy [31].

#### 3.8.3 Efficacy‐associated PIK3CA mutation sites reported

In the SOLAR‐1 trial, 11 common mutation sites of PIK3CA were tested via QIAGEN therascreen®PIK3CA RGQ PCR kit, including mutation in exons 7, 9, and 20 (C420R, E542K, E545A/D/G/K, Q546E/R, and H1047L/R/Y) [84]. For those with one or more PIK3CA mutations in either tissue or plasma specimen, alpelisib therapy can be administered [86]. Furthermore, common mutation sites on PIK3CA, including E545G, E545K, Q546E, Q546R, H1047L, H1047R, and H1047Y, can also be detected by F1LCDx, an NGS‐based liquid biopsy system [87]. A real‐world study by ASCO published in 2021 reported that patients with N345K, Q75E, R38C, G106_108del, and N345K/N1044K mutations in PIK3CA can also benefit from alpelisib [88]. With NGS technologies, more mutation sites can be detected, but the correlations between new mutation sites and drug efficacy still need to be determined.

#### 3.8.4 Commonly used methods for PIK3CA mutation testing

Currently, commonly used methods for PIK3CA mutation testing include fluorescent dye‐based real‐time PCR (RT‐PCR) and NGS. Compared to RT‐PCR, more mutation sites can be detected with NGS. Other methods include droplet digital PCR (ddPCR), BEAMing, and Sanger sequencing [79, 89, 90]. With alpelisib approved by FDA, as an accompanying product for diagnosis, the QIAGEN therascreen®PIK3CA RGQ PCR kit also became available for PIK3CA mutation testing with tissue and/or peripheral blood ctDNA (liquid biopsy) specimens [14]. Then, F1LCDx, an NGS‐based accompanying product for liquid biopsy, is also approved by FDA for the detection of PIK3CA mutations [87]. Each testing method has its own advantages and disadvantages, with test results affected by detection limit, type of specimen, quantity, quality of the specimen, and so forth.

Expert panel recommendations: PIK3CA mutation testing is recommended for patients with HR‐positive/HER2‐negative advanced or metastatic breast cancer who exhibited disease progression after endocrine‐based therapy. As there is not yet a national standard for mutation testing, either medical centers with the capability of genetic testing or fully certified genetic testing laboratories should be selected for PIK3CA mutation testing. All testing procedures in the laboratories should be approved by authorities, to ensure the accuracy of test results. Either tumor tissue or plasma ctDNA can be collected for PIK3CA mutation testing, but fresh, paraffin‐embedded tumor tissue is preferred. If unavailable, previously harvested paraffin‐embedded tumor tissue or slices, as well as plasma, can also be considered. If PIK3CA mutation is not detected in plasma ctDNA specimen, it is recommended to harvest tumor tissue specimen to further clarify the PIK3CA mutation status. Due to the similar distribution of PIK3CA mutation in primary and metastatic sites, either fresh biopsy tissue samples or archived tissue samples can be used for mutation testing. It is also recommended that relevant mutation sites of PIK3CA reported by the SOLAR‐1 trial and real‐world studies be tested, such as mutations in exons 7, 9, and 20 (C420R, E542K, E545A/D/G/K, Q546E/R, and H1047L/R/Y), and so forth. For other mutation sites, there is still no clear evidence supporting their association with clinical efficacy, and further studies are needed.

## 4 FUTURE PERSPECTIVES

In previous studies, PAM pathway inhibitors were mainly examined as a first‐line or late‐line treatment for HR‐positive/HER2‐negative advanced or metastatic breast cancer. However, aberrant activation of PAM pathway is not only a potential mechanism for resistance to endocrine therapy and CDK4/6 inhibitors but is also closely associated with resistance to anti‐HER2 therapy and chemotherapy. Therefore, the application of PAM pathway inhibitors in triple‐negative breast cancer and HER2‐positive breast cancer is currently being explored.

### 4.1 PI3K inhibitors

Outstanding efficacy and safety profiles have been reported on alpelisib in patients with PIK3CA‐mutated, HR‐positive/HER2‐negative advanced breast cancer, and some efficacy has also been shown in triple‐negative breast cancer and HER2‐positive breast cancer. The NCT02038010 study [91] found that good tolerance and preliminary efficacy were achieved by combination therapy of alpelisib plus T‐DM1 in trastuzumab‐refractory, HER2‐positive metastatic breast cancer, laying the foundation for further studies. Moreover, a phase III trial (CBYL719C2201/NCT04544189) on alpelisib plus fulvestrant in Chinese patients with PIK3CA‐mutated, HR‐positive/HER2‐negative breast cancer who previously received AI therapy, a phase III trial (CBYL719H12301/NCT04251533) on alpelisib plus nab‐paclitaxel in triple‐negative breast cancer patients with PIK3CA mutation or with PTEN loss but without PIK3CA mutation, and a phase III trial (CBYL719G12301/NCT04208178) on alpelisib plus trastuzumab and pertuzumab as maintenance therapy in patients with PIK3CA‐mutated, HER2‐positive advanced breast cancer are still underway.

Other PI3K inhibitors, such as taselisib, eganelisib, and MEN1611, are still being explored in the treatment of breast cancer. The LORELEI study has proved for the first time that compared to letrozole monotherapy, taselisib plus letrozole can significantly improve the ORR in patients with ER‐positive/HER2‐negative early‐stage breast cancer, supporting its role in overcoming the resistance to neoadjuvant endocrine therapy [92]. The MARIO‐3 study provided preliminary evidence supporting the efficacy and safety of the combination therapy of eganelisib, tezolizumab, and chemotherapy in unresectable, triple‐negative locally advanced, or metastatic breast cancer. Good tolerance was also observed from the combination therapy of MEN1611 and trastuzumab with or without fulvestrant in PIK3CA‐mutated, HER2‐positive advanced or metastatic breast cancer [93].

### 4.2 AKT inhibitors

Besides ipatasertib and capivasertib, other AKT inhibitors under research include MK‐2206, vevorisertib, and so forth, but no positive clinical data have been obtained.

### 4.3 mTOR inhibitors

Combination therapy of everolimus plus endocrine therapy in patients with HR‐positive/HER2‐negative advanced or metastatic breast cancer after treatment failure with endocrine therapy has been established in clinical practice and included in many guidelines. Multiple real‐world studies have observed the advantages of this combination therapy over other endocrine therapies in patients with advanced breast cancer who had experienced disease progression with CDK4/6 inhibitors. This combination therapy is also recommended by international guidelines as well as Chinese guidelines in this population.

The anti‐HER2 effects of PAM pathway inhibitors have also been predicted by early‐phase studies, including BOLERO‐1 and BOLERO‐3 trials. In the BOLERO‐3 trial, PFS in patients with HER2‐positive breast cancer was significantly improved in the group treated with everolimus plus trastuzumab plus vinorelbine, in comparison to placebo plus trastuzumab plus vinorelbine. Although the effects of everolimus on PFS in BOLERO‐1 did not reach the preset significant level in the overall analysis, median PFS in ER‐negative patients turned out to be 7.2 months longer than in the control group (Table 14).

**Table 14:** Clinical studies related to PAM pathway inhibitors

Target	Study	Number of cases	Treatment regimen	Median PFS (months)	Hazrd ratio (HR) of PFS
**ER‐positive/HER2‐negative early‐stage breast cancer**					
PI3K	LORELEI [92]	334	Taselisib + letrozole; placebo + letrozole	‐	‐
**ER‐positive advanced breast cancer**					
PI3K	NCT01872260 [96]	‐	LEE011 (a CDK4/6 inhibitor) + letrozole; alpelisib + letrozole; LEE011 + letrozole + alpelisib	‐	‐
**Triple‐negative advanced breast cancer with PIK3CA mutation or PTEN loss**					
PI3K	CBYL719H12301 (EPIK‐B3) [97]	566	Alpelisib + nab‐paclitaxel; placebo + nab‐paclitaxel (As first‐ or second‐line treatment)	‐	‐
PI3K	MARIO‐3 [93]	90	Eganelisib (IPI‐549) + atezolizumab + nab‐palitaxel	‐	‐
**PIK3CA‐mutated, HER2‐positive advanced breast cancer**					
PI3K	CBYL719G12301(EPIK‐B2) [98]	‐	Alpelisib (BYL719) + trastuzumab + pertuzumab; placebo + trastuzumab + pertuzumab (maintenance therapy)	‐	‐
PI3K	B‐PRECISE‐01	‐	MEN1611 + trastuzumab ± fulvestrant	‐	‐
**ER‐negative/HER2‐positive advanced breast cancer**					
mTOR	BOLERO‐1 [99]	‐	Everolimus + trastuzumab + paclitaxel; placebo + trastuzumab + paclitaxel	20.27; 13.08	0.66
**HER2‐positive advanced breast cancer**					
mTOR	BOLERO‐3 [100]	572	Everolimus + trastuzumab + Vinorelbine; placebo + trastuzumab + Vinorelbine	7.00; 5.78	0.78

Other mTOR inhibitors under investigation include sapanisertib and vistusertib. The Phase II trial of sapanisertib and fulvestrant combination therapy failed to show any actual improvement in prognosis in postmenopausal female patients with ER‐positive/HER‐negative advanced breast cancer after disease progression with AI therapy [94]. In the MANTA study, no significant difference in PFS was found between vistusertib plus fulvestrant group and the fulvestrant monotherapy group [95].

## 5 CONCLUSION

Currently, although combination therapy of endocrine therapy plus CDK4/6 inhibitors remains the standard first‐line treatment for patients with HR‐positive/HER2‐negative advanced breast cancer, drug resistance still needs to be addressed [3, 6]. The PAM pathway plays an essential role in cell growth, survival, proliferation, angiogenesis, as well as resistance to endocrine therapy and CDK4/6 inhibitors in breast cancer, making it an important treatment target. As of now, many PAM pathway inhibitors are still under development, which might potentially improve the management of breast cancer [6, 12, 13]. Those that have already been approved, including a PI3K inhibitor (alpelisib) and an mTOR inhibitor (everolimus), provide more options for patients with HR‐positive/HER2‐negative advanced breast cancer after disease progression with endocrine therapy. In breast cancer of other molecular subtypes, PAM pathway inhibitors also showed some preliminary efficacy, indicating that more patients might benefit from PAM pathway inhibitors in the future.

## AUTHOR CONTRIBUTIONS

**Fei Ma**: conceptualization (equal); data curation (equal); formal analysis (equal); funding acquisition (equal); investigation (equal); methodology (equal); project administration (equal); resources (equal); software (equal); supervision (equal); validation (equal); visualization (equal); writing–original draft (equal); writing–review & editing (equal). **Binghe Xu**: conceptualization (equal); data curation (equal); formal analysis (equal); funding acquisition (equal); investigation (equal); methodology (equal); project administration (equal); resources (equal); software (equal); supervision (equal); validation (equal); visualization (equal); writing–original draft (equal); writing–review & editing (equal).

## CONFLICTS OF INTEREST

All authors declare that there is no conflict of interest except Professor Fei Ma, Professor Binghe Xu, Professor Haili Qian, and Professor Jiuda Zhao, who are members of the Cancer Innovation Editorial Board. To minimize bias, they were excluded from all editorial decision‐making related to the acceptance of this article for publication.

## ETHICS STATEMENT

Not applicable.

## INFORMED CONSENT

Not applicable.

## Data Availability

Data sharing is not applicable to this article as no new data were created or analyzed in this study.

## References

[1] Sung H , Ferlay J , Siegel RL , Laversanne M , Soerjomataram I , Jemal A , et al. Global cancer statistics 2020: globocan estimates of incidence and mortality worldwide for 36 cancers in 185 countries. CA Cancer J Clin. 2021;71(3):209–49. 10.3322/caac.21660 33538338

[2] Cao W , Chen HD , Yu YW , et al. Changing profiles of cancer burden worldwide and in China: a secondary analysis of the global cancer statistics 2020. Chin Med J (Engl). 2021;134(7):783–91. 10.1097/CM9.0000000000001474 33734139 PMC8104205

[3] Harbeck N , Penault‐Llorca F , Cortes J , Gnant M , Houssami N , Poortmans P , et al. Breast cancer. Nat Rev Dis Primers. 2019;5(1):66. 10.1038/s41572-019-0111-2 31548545

[4] Lim E , Metzger‐Filho O , Winer EP . The natural history of hormone receptor‐positive breast cancer. Oncology (Williston Park). 2012;26(8):688–94. 22957400

[5] Dong C , Wu J , Chen Y , Nie J , Chen C . Activation of PI3K/AKT/mTOR pathway causes drug resistance in breast cancer. Front Pharmacol. 2021;12:628690. 10.3389/fphar.2021.628690 33790792 PMC8005514

[6] Nunnery SE , Mayer IA . Targeting the PI3K/AKT/mTOR pathway in hormone‐positive breast cancer. Drugs. 2020;80(16):1685–97. 10.1007/s40265-020-01394-w 32894420 PMC7572750

[7] Ciruelos Gil EM . Targeting the PI3K/AKT/mTOR pathway in estrogen receptor‐positive breast cancer. Cancer Treat Rev. 2014;40(7):862–71. 10.1016/j.ctrv.2014.03.004 24774538

[8] Burris HA 3rd . Overcoming acquired resistance to anticancer therapy: focus on the PI3K/AKT/mTOR pathway. Cancer Chemother Pharmacol. 2013;71(4):829–42. 10.1007/s00280-012-2043-3 23377372

[9] Sharma VR , Gupta GK , Sharma AK , Batra N , Sharma DK , Joshi A , et al. PI3K/AKT/mTOR intracellular pathway and breast cancer: factors, mechanism and regulation. CurrPharmDes. 2017;23(11):1633–38. 10.2174/1381612823666161116125218 27848885

[10] Dienstmann R , Rodon J , Serra V , et al. Picking the point of inhibition: a comparative review of PI3K/AKT/mTOR pathway inhibitors. Mol Cancer Ther. 2014;13(5):1021–31. 10.1158/1535-7163 24748656

[11] Brufsky AM , Dickler MN . Estrogen receptor‐positive breast cancer: exploiting signaling pathways implicated in endocrine resistance. Oncologist. 2018;23(5):528–39. 10.1634/theoncologist.2017-0423 29352052 PMC5947450

[12] O'Brien NA , McDermott MSJ , Conklin D , et al. Targeting activated PI3K/mTOR signaling overcomes acquired resistance to CDK4/6‐based therapies in preclinical models of hormone receptor‐positive breast cancer. Breast Cancer Res. 2020;22(1):89. 10.1186/s13058-020-01320-8 32795346 PMC7427086

[13] Li H , Prever L , Hirsch E , Gulluni F . Targeting PI3K/AKT/mTOR signaling pathway in breast cancer. Cancers (Basel). 2021;13(14):3517. 10.3390/cancers13143517 34298731 PMC8304822

[14] Novartis Pharmaceuticals Corporation . Highlights of Prescribing Information, PIQRAY® (Alpelisib) tablets, for oral use, Initial U.S. [EB/OL]. (2019‐05) [2022‐05‐10]. https://www.accessdata.fda.gov/drugsatfda_docs/label/2019/212526s000lbl.pdf

[15] Jia M , Liao N , Chen B , Zhang G , Wang Y , Li X , et al. PIK3CA somatic alterations in invasive breast cancers: different spectrum from Caucasians to Chinese detected by next generation sequencing. Breast Cancer. 2021;28(3):644–52. 10.1007/s12282-020-01199-5 33386585 PMC8065000

[16] Novartis Pharmaceuticals Corporation . Highlights of Prescribing Information, AFINITOR® (everolimus) tablets for oral administration, AFINITOR® DISPERZ (everolimus tablets for oral suspension), Initial U.S. [EB/OL]. (2014‐07) [2022‐05‐10]. https://www.novartis.us/sites/www.novartis.us/files/afinitor.pdf?irmasrc=ONCWB0043%26source=01030.pdf.

[17] Yardley DA , Noguchi S , Pritchard KI , Burris HA , Baselga J , Gnant M , et al. Everolimus plus exemestane in postmenopausal patients with HR (+) breast cancer: BOLERO‐2 final progression‐free survival analysis. Adv Ther. 2013;30(10):870–84. 10.1007/s12325-013-0060-1 24158787 PMC3898123

[18] Kornblum N , Zhao F , Manola J , Klein P , Ramaswamy B , Brufsky A , et al. Randomized phase II trial of fulvestrant plus everolimus or placebo in postmenopausal women with hormone receptor‐positive, human epidermal growth factor receptor 2‐negative metastatic breast cancer resistant to aromatase inhibitor therapy: results of PrE0102. J Clin Oncol. 2018;36(16):1556–63. 10.1200/JCO.2017.76.9331 29664714 PMC7186582

[19] Shao Z , Cai L , Wang S , Hu X , Shen K , Wang H , et al. 2021. BOLERO‐5: a phase II study of everolimus and exemestance combination in Chinese postmenopausal women with ER+/HER2‐ advanced breast cancer. ESMO POSTER 238P. 10.1007/s12672-024-01027-8 PMC11192707 38904918

[20] Royce M , Bachelot T , Villanueva C , Özgüroglu M , Azevedo SJ , Cruz FM , et al. Everolimus plus endocrine therapy for postmenopausal women with estrogen receptor‐positive, human epidermal growth factor receptor 2‐negative advanced breast cancer: a clinical trial. JAMA Oncol. 2018;4(7):977–84. 10.1001/jamaoncol.2018.0060 29566104 PMC5885212

[21] Fan Y , Sun T , Shao Z , Zhang Q , Ouyang Q , Tong Z , et al. Effectiveness of adding everolimus to the first‐line treatment of advanced breast cancer in premenopausal women who experienced disease progression while receiving selective estrogen receptor modulators: a phase 2 randomized clinical trial. JAMA Oncol. 2021;7(10):e213428. 10.1001/jamaoncol.2021.3428 34436536 PMC8391779

[22] Li Y , Li W , Gong C , Zheng Y , Ouyang Q , Xie N , et al. A multicenter analysis of treatment patterns and clinical outcomes of subsequent therapies after progression on palbocicli/HER2‐ metastatic breast cancer. Ther Adv Med Oncol. 2021;13:17588359211022890. 10.1177/17588359211022890 34178122 PMC8202336

[23] Cidado J , Park BH . Targeting the PI3K/Akt/mTOR pathway for breast cancer therapy. J Mammary Gland Biol Neoplasia. 2012;17(3–4):205–16. 10.1007/s10911-012-9264-2 22865098 PMC3724399

[24] McKenna M , McGarrigle S , Pidgeon GP . The next generation of PI3K‐Akt‐mTOR pathway inhibitors in breast cancer cohorts. Biochim Biophys Acta, Rev Cancer. 2018;1870(2):185–97. 10.1016/j.bbcan.2018.08.001 30318472

[25] Miricescu D , Totan A , Stanescu‐Spinu II , Badoiu SC , Stefani C , Greabu M . PI3K/AKT/mTOR signaling pathway in breast cancer: from molecular landscape to clinical aspects. Int J Mol Sci. 2020;22(1):173. 10.3390/ijms22010173 33375317 PMC7796017

[26] Lee JJ , Loh K , Yap YS . PI3K/Akt/mTOR inhibitors in breast cancer. Cancer Biol Med. 2015;12(4):342–54. 10.7497/j.issn.2095-3941.2015.0089 26779371 PMC4706528

[27] Presti D , Quaquarini E . The PI3K/AKT/mTOR and CDK4/6 pathways in endocrine resistant HR+/HER2‐ metastatic breast cancer: biological mechanisms and new treatments. Cancers (Basel). 2019;11(9):1242. 10.3390/cancers11091242 31450618 PMC6770492

[28] Wilks ST . Potential of overcoming resistance to HER2‐targeted therapies through the PI3K/Akt/mTOR pathway. Breast. 2015;24(5):548–55. 10.1016/j.breast.2015.06.002 26187798

[29] Gao C , Yuan X , Jiang Z , Gan D , Ding L , Sun Y , et al. Regulation of AKT phosphorylation by GSK3β and PTEN to control chemoresistance in breast cancer. Breast Cancer Res Treat. 2019;176(2):291–301. 10.1007/s10549-019-05239-3 31006103

[30] André F , Ciruelos E , Rubovszky G , Campone M , Loibl S , Rugo HS , et al. alpelisib for PIK3CA‐mutated, hormone receptor‐positive advanced breast cancer. N Engl J Med. 2019;380(20):1929–40. 10.1056/NEJMoa1813904 31091374

[31] Juric D , Ciruelos E , Rubovszky G , Campone M , Loibl S , Rugo HS , et al. Alpelisib (ALP)+fulvestrant (FUL) for advanced breast cancer (ABC): phase 3 SOLAR‐1 trial results. 2018 SABCS. Abstract GS3‐08 (oral).

[32] André F , Ciruelos EM , Juric D , Loibl S , Campone M , Mayer IA , et al. Alpelisib plus fulvestrant for PIK3CA‐mutated, hormone receptor‐positive, human epidermal growth factor receptor‐2‐negative advanced breast cancer: final overall survival results from SOLAR‐1. Ann Oncol. 2021;32(2):208–17. 10.1016/j.annonc.2020.11.011 33246021

[33] Rugo HS , Lerebours F , Ciruelos E , Drullinsky P , Ruiz‐Borrego M , Neven P , et al. Alpelisib plus fulvestrant in PIK3CA‐mutated, hormone receptor‐positive advanced breast cancer after a CDK4/6 inhibitor (BYLieve): one cohort of a phase 2, multicentre, open‐label, non‐comparative study. Lancet Oncol. 2021;22(4):489–98. 10.1016/S1470-2045(21)00034-6 33794206

[34] Rugo HS , Lerebours F , Juric D , Turner N , Chia S , Drullinsky P , et al. Alpelisib + letrozole in patients with PIK3CA‐mutated, hormone receptor‐positive (HR+), human epidermal growth factor receptor 2‐negative (HER2–) advanced breast cancer (ABC) previously treated with a cyclin‐dependent kinase 4/6 inhibitor (CDK4/6i) + fulvestrant: BYLieve study results. 2020 SABCS. Abstract PD2‐07 (poster).

[35] Chia S , Ruiz‐Borrego M , Drullinsky P , Juric D , Bachelot T , Rugo HS , et al. Impact of duration of prior cyclin‐dependent kinase 4/6 inhibitor (CDK4/6i) therapy on alpelisib (ALP) benefit in patients (pts) with hormone receptor–positive (HR+), human epidermal growth factor receptor‐2–negative (HER2–), PIK3CA‐mutated advanced breast cancer (ABC) from BYLieve. ASCO. Abstract 1060 (poster). 2021.

[36] Rugo HS , Neven P , Saffie I , Park YH , De Laurentiis M , Lerebours F , et al. Alpelisib + fulvestrant in patients with PIK3CA‐mutated, HR+, HER2– advanced breast cancer (ABC) who received chemotherapy or endocrine therapy (ET) as immediate prior treatment: BYLieve cohort c primary results and exploratory biomarker analyses. 2021. SABCS. Poster PD13‐05.

[37] Lu YS , Lee KS , Chao TY , Tseng LM , Chitapanarux I , Chen SC , et al. A phase ib study of alpelisib or buparlisib combined with tamoxifen plus goserelin in premenopausal women with HR‐positive HER2‐negative advanced breast cancer. Clin Cancer Res. 2021;27(2):408–17. 10.1158/1078-0432.CCR-20-1008 32718997

[38] National Comprehensive Cancer Network . NCCN clinical practical guidelines in oncology breast cancer. Version 2. [EB/OL]. (2021‐12‐20) [2022‐05‐10]. https://www.nccn.org/.

[39] Advanced Breast Cancer Sixth International Consensus Conference . 4‐6 November 2021.

[40] Breast Cancer Professional Committee of Chinese Anti‐Cancer Society . Chinese Association of Cancer Diagnosis and Treatment guidelines for Breast cancer (2021 edition). Chin J Cancer. 2021;31(10):954–1040. 10.19401/j.cnki.1007-3639.2021.10.013

[41] Bedard PL , Jhaveri K , Cervantes A , Gambardella V , Hamilton E , Italiano A , et al. A phase I/Ib study evaluating GDC‐0077 + palbociclib (palbo) + fulvestrant in patients (pts) with PIK3CA‐mutant (mut), hormone receptor‐positive/HER2‐negative metastatic breast cancer (HR+/HER2‐ mBC). 2020. SABCS. Poster PD1‐2.

[42] Jhaveri K , Juric D , Varga A , Turner N , Schmid P , Saura C , et al. Preliminary correlative analysis of clinical outcomes with PIK3CA mutation (mut) status from a phase I/Ib study of GDC‐0077 in patients (pts) with hormone receptor‐positive/HER2‐negative metastatic breast cancer (HR+/HER2‐mBC). 2020. SABCS. Poster PS5‐12.

[43] Juric D , Bedard PL , Cervantes A , Gambardella V , Oliveira M , Saura C , et al. A phase I/Ib study of inavolisib (GDC‐0077) in combination with fulvestrant in patients (pts) with PIK3CA‐mutated hormone receptor‐positive/HER2‐negative (HR+/HER2–) metastatic breast cancer. 2021 SABCS. Poster P5‐17‐05.

[44] Dent R , Kim SB , Oliveira M , Barrios C , O'Shaughnessy J , Isakoff SJ , et al. Double‐blind placebo (PBO)‐controlled randomized phase III trial evaluating first‐line ipatasertib (IPAT) combined with paclitaxel (PAC) for PIK3CA/AKT1/PTEN‐altered locally advanced unresectable or metastatic triple‐negative breast cancer (aTNBC): primary results from IPATunity130 Cohort A. SABCS 2020. GS3‐04.

[45] Jones RH , Casbard A , Carucci M , Cox C , Butler R , Alchami F , et al. Fulvestrant plus capivasertib versus placebo after relapse or progression on an aromatase inhibitor in metastatic, oestrogen receptor‐positive breast cancer (FAKTION): a multicentre, randomised, controlled, phase 2 trial. Lancet Oncol. 2020;21(3):345–57. 10.1016/S1470-2045(19)30817-4 32035020 PMC7052734

[46] Smyth LM , Tamura K , Oliveira M , Ciruelos EM , Mayer IA , Sablin MP , et al. Capivasertib, an AKT kinase inhibitor, as monotherapy or in combination with fulvestrant in patients with AKT1E17K‐mutant, ER‐positive metastatic breast cancer. Clin Cancer Res. 2020;26(15):3947–57. 10.1158/1078-0432.CCR-19-3953 32312891 PMC7415507

[47] Piccart M , Hortobagyi GN , Campone M , Pritchard KI , Lebrun F , Ito Y , et al. Everolimus plus exemestane for hormone‐receptor‐positive, human epidermal growth factor receptor‐2‐negative advanced breast cancer: overall survival results from BOLERO‐2†. Ann Oncol. 2014;25(12):2357–62. 10.1093/annonc/mdu456 25231953 PMC6267855

[48] Bachelot T , Bourgier C , Cropet C , Ray‐Coquard I , Ferrero JM , Freyer G , et al. Randomized phase II trial of everolimus in combination with tamoxifen in patients with hormone receptor‐positive, human epidermal growth factor receptor 2‐negative metastatic breast cancer with prior exposure to aromatase inhibitors: a GINECO study. J Clin Oncol. 2012;30(22):2718–24. 10.1200/JCO.2011.39.0708 22565002

[49] Bardia A , Hurvitz SA , DeMichele A , Clark AS , Zelnak A , Yardley DA , et al. Phase I/II trial of exemestane, ribociclib, and everolimus in women with HR+/HER2‐ advanced breast cancer after progression on CDK4/6 inhibitors (TRINITI‐1). Clin Cancer Res. 2021;27(15):4177–85. 10.1158/1078-0432.CCR-20-2114 33722897 PMC8487593

[50] Xi J , Oza A , Thomas S , Ademuyiwa F , Weilbaecher K , Suresh R , et al. Retrospective analysis of treatment patterns and effectiveness of palbociclib and subsequent regimens in metastatic breast cancer. J Natl Compr Canc Netw. 2019;17(2):141–7. 10.6004/jnccn.2018.7094 30787127 PMC6752198

[51] Yi Z , Liu B , Sun X , Rong G , Wang W , Li H , et al. Safety and efficacy of sirolimus combined with endocrine therapy in patients with advanced hormone receptor‐positive breast cancer and the exploration of biomarkers. Breast. 2020;52:17–22. 10.1016/j.breast.2020.04.004 32335491 PMC7375615

[52] Novartis Pharmaceuticals Corporation . Highlights of Prescribing Information, PIQRAY® (Alpelisib) tablets, for oral use, Initial HK. [EB/OL]. (2020‐11‐05) [2022‐05‐10].

[53] Kirchner GI , Meier‐Wiedenbach I , Manns MP . Clinical pharmacokinetics of everolimus. Clin Pharmacokinet. 2004;43(2):83–95. 10.2165/00003088-200443020-00002 14748618

[54] Huang X , Liu G , Guo J , Su Z . The PI3K/AKT pathway in obesity and type 2 diabetes. Int J Biol Sci. 2018;14(11):1483–96. 10.7150/ijbs.27173 30263000 PMC6158718

[55] Goldman JW , Mendenhall MA , Rettinger SR . Hyperglycemia associated with targeted oncologic treatment: mechanisms and management. Oncologist. 2016;21(11):1326–36. 10.1634/theoncologist.2015-0519 27473045 PMC5189614

[56] Goncalves MD , Hopkins BD , Cantley LC . Phosphatidylinositol 3‐kinase, growth disorders, and cancer. N Engl J Med. 2018;379(21):2052–62. 10.1056/NEJMra1704560 30462943

[57] Fruman DA , Chiu H , Hopkins BD , Bagrodia S , Cantley LC , Abraham RT . The PI3K pathway in human disease. Cell. 2017;170(4):605–35. 10.1016/j.cell.2017.07.029 28802037 PMC5726441

[58] Rugo HS , André F , Yamashita T , Cerda H , Toledano I , Stemmer SM , et al. Time course and management of key adverse events during the randomized phase III SOLAR‐1 study of PI3K inhibitor alpelisib plus fulvestrant in patients with HR‐positive advanced breast cancer. Ann Oncol. 2020;31(8):1001–10. 10.1016/j.annonc.2020.05.001 32416251

[59] Rugo HS , Lacouture ME , Goncalves MD , Masharani U , Aapro MS , O'Shaughnessy JA . A multidisciplinary approach to optimizing care of patients treated with alpelisib. Breast. 2022;61:156–67. 10.1016/j.breast.2021.12.016 35016012 PMC8749445

[60] Baselga J , Campone M , Piccart M , Burris HA , Rugo HS , Sahmoud T , et al. Everolimus in postmenopausal hormone‐receptor‐positive advanced breast cancer. N Engl J Med. 2012;366(6):520–9. 10.1056/NEJMoa1109653 22149876 PMC5705195

[61] Gong C , Xiao Q , Li Y , Gu Y , Zhang J , Wang L , et al. Everolimus‐related pneumonitis in patients with metastatic breast cancer: incidence, radiographic patterns, and relevance to clinical outcome. Oncologist. 2021;26(4):e580–87. 10.1002/onco.13594 33191524 PMC8018320

[62] Rugo HS , Pritchard KI , Gnant M , Noguchi S , Piccart M , Hortobagyi G , et al. Incidence and time course of everolimus‐related adverse events in postmenopausal women with hormone receptor‐positive advanced breast cancer: insights from BOLERO‐2. Ann Oncol. 2014;25(4):808–15. 10.1093/annonc/mdu009 24615500 PMC3969554

[63] Seiler S , Kosse J , Loibl S , Jackisch C . Adverse event management of oral mucositis in patients with breast cancer. Breast Care (Basel). 2014;9(4):232–7. 10.1159/000366246 25404881 PMC4209263

[64] Rugo HS , Seneviratne L , Beck JT , Glaspy JA , Peguero JA , Pluard TJ , et al. Prevention of everolimus‐related stomatitis in women with hormone receptor‐positive, HER2‐negative metastatic breast cancer using dexamethasone mouthwash (SWISH): a single‐arm, phase 2 trial. Lancet Oncol. 2017;18(5):654–62. 10.1016/S1470-2045(17)30109-2 28314691

[65] Aapro M , Andre F , Blackwell K , Calvo E , Jahanzeb M , Papazisis K , et al. Adverse event management in patients with advanced cancer receiving oral everolimus: focus on breast cancer. Ann Oncol. 2014;25(4):763–73. 10.1093/annonc/mdu021 24667713

[66] Duran I , Goebell PJ , Papazisis K , Ravaud A , Weichhart T , Rodriguez‐Portal JA , et al. Drug‐induced pneumonitis in cancer patients treated with mTOR inhibitors: management and insights into possible mechanisms. Expert Opin Drug Saf. 2014;13(3):361–72. 10.1517/14740338.2014.888056 24517115

[67] Working Committee of Respiratory Pathology, Branch of Respiratory Physicians, Chinese Medical Association . Chinese expert consensus on pathologic diagnosis of common interstitial pneumonia (draft). China J Tuberculosis Respiratory. 2018;41(3):186–90.

[68] Everolimus Clinical Safety Management Expert Panel . Expert opinion on clinical safety management of everolimus. China New Drug J. 2014;(22):2694–700.

[69] Peddi PF , Shatsky RA , Hurvitz SA . Noninfectious pneumonitis with the use of mTOR inhibitors in breast cancer. Cancer Treat Rev. 2014;40(2):320–6. 10.1016/j.ctrv.2013.08.004 24011786

[70] Samuels Y , Wang Z , Bardelli A , Silliman N , Ptak J , Szabo S , et al. High frequency of mutations of the PIK3CA gene in human cancers. Science. 2004;304(5670):554. 10.1126/science.1096502 15016963

[71] Courtney KD , Corcoran RB , Engelman JA . The PI3K pathway as drug target in human cancer. J Clin Oncol. 2010;28(6):1075–83. 10.1200/JCO.2009.25.3641 20085938 PMC2834432

[72] Mukohara T . PI3K mutations in breast cancer: prognostic and therapeutic implications. Breast Cancer. 2015;7:111–23. 10.2147/BCTT.S60696 26028978 PMC4440424

[73] Isakoff SJ , Engelman JA , Irie HY , Luo J , Brachmann SM , Pearline RV , et al. Breast cancer‐associated PIK3CA mutations are oncogenic in mammary epithelial cells. Cancer Res. 2005;65(23):10992–1000. 10.1158/0008-5472.CAN-05-2612 16322248

[74] Bachman KE , Argani P , Samuels Y , Silliman N , Ptak J , Szabo S , et al. The PIK3CA gene is mutated with high frequency in human breast cancers. Cancer Biol Ther. 2004;3(8):772–5. 10.4161/cbt.3.8.994 15254419

[75] Campbell IG , Russell SE , Choong DY , Montgomery KG , Ciavarella ML , Hooi CS , et al. Mutation of the PIK3CA gene in ovarian and breast cancer. Cancer Res. 2004;64(21):7678–81. 10.1158/0008-5472.CAN-04-2933 15520168

[76] Levine DA , Bogomolniy F , Yee CJ , Lash A , Barakat RR , Borgen PI , et al. Frequent mutation of the PIK3CA gene in ovarian and breast cancers. Clin Cancer Res. 2005;11(8):2875–8. 10.1158/1078-0432.CCR-04-2142 15837735

[77] Lee JW , Soung YH , Kim SY , Lee HW , Park WS , Nam SW , et al. PIK3CA gene is frequently mutated in breast carcinomas and hepatocellular carcinomas. Oncogene. 2005;24(8):1477–80. 10.1038/sj.onc.1208304 15608678

[78] Chen L , Yang L , Yao L , Kuang XY , Zuo WJ , Li S , et al. Characterization of PIK3CA and PIK3R1 somatic mutations in Chinese breast cancer patients. Nat Commun. 2018;9(1):1357. 10.1038/s41467-018-03867-9 29636477 PMC5893593

[79] Martínez‐Sáez O , Chic N , Pascual T , Adamo B , Vidal M , González‐Farré B , et al. Frequency and spectrum of PIK3CA somatic mutations in breast cancer. Breast Cancer Res. 2020;22(1):45. 10.1186/s13058-020-01284-9 32404150 PMC7222307

[80] Yi Z , Ma F , Li C , Chen R , Yuan L , Sun X , et al. Landscape of somatic mutations in different subtypes of advanced breast cancer with circulating tumor DNA analysis. Sci Rep. 2017;7(1):5995. 10.1038/s41598-017-06327-4 28729728 PMC5519668

[81] Deng L , Zhu X , Sun Y , Wang J , Zhong X , Li J , et al. Prevalence and prognostic role of PIK3CA/AKT1 mutations in chinese breast cancer patients. Cancer Res Treat. 2019;51(1):128–40. 10.4143/crt.2017.598 29540052 PMC6333988

[82] Burstein HJ , Somerfield MR , Barton DL , Dorris A , Fallowfield LJ , Jain D , et al. Endocrine treatment and targeted therapy for hormone receptor‐positive, human epidermal growth factor receptor 2‐negative metastatic breast cancer: ASCO Guideline Update. J Clin Oncol. 2021;39(35):3959–77. 10.1200/JCO.21.01392 34324367 PMC8659999

[83] Cardoso F , Paluch‐Shimon S , Senkus E , Curigliano G , Aapro MS , André F , et al. 5th ESO‐ESMO international consensus guidelines for advanced breast cancer (ABC 5). Ann Oncol. 2020;31(12):1623–49. 10.1016/j.annonc.2020.09.010 32979513 PMC7510449

[84] Ciruelos EM , Loibl S , Mayer IA , Campone M , Rugo HS , Arnedos M , et al. Clinical outcome of alpelisib plus fulvestrant in hormone receptor‐positive, human epidermal growth factor receptor 2‐negative advanced breast cancer with PIK3CA alterations detected in plasma ctDNA by next‐generation sequencing: biomarker analysis from the solar‐1 study. 2020 SABCS. PD2‐06.

[85] Rugo H , Mayer I , Conte P , Loibl S , Campone M , Juric D , et al. Prevalence of PIK3CAmutations in patients with hormone receptor‐positive, human epidermal growth factor‐2‐negative advanced breast cancer from the SOLAR‐1 trial. 2019. AACR. Abstract CT142.

[86] QIAGEN GmbH . therascreen® PIK3CA RGQ PCR Kit Instructions for Use (Handbook). [EB/OL]. (2019‐05) [2022‐05‐10]. https://www.accessdata.fda.gov/scripts/cdrh/cfdocs/cfpma/pma.cfm?id=P190001.

[87] U.S. FOOD&DRUG ADMINISTRATION . Premarket Approval (PMA), FoundationOne Liquid CDx (F1 Liquid CDx). https://www.accessdata.fda.gov/scripts/cdrh/cfdocs/cfpma/pma.cfm?id=P200006. [EB/OL].

[88] Rugo HS , Raskina K , Schrock AB , Israel MA , Sokol E , Sivakumar S , et al. Real‐world (rw) clinical outcomes on alpelisib (ALP) in patients (pts) with breast cancer (BC) and PIK3CA mutations (PIK3CAm). 2021. ASCO. Abstract 1068.

[89] Juric D , Andre F , Singer CF , Sohn J , Campone M , Loibl S , et al. Clinical outcomes of alpelisib in hormone receptor‐positive, human epidermal growth factor receptor‐2‐negative advanced breast cancer by next‐generation sequencing‐detected PIK3CA alteration status and phosphatase and tensin homolog loss: biomarker analysis from the SOLAR‐1 study. 2019.

[90] My Cancer Genome® , GENETICALLY INFORMED CANCER MEDICINE. Types of Molecular Tumor Testing. [EB/OL]. (2014‐06) [2022‐05‐10]. https://www.mycancergenome.org/content/page/molecular-testing

[91] Jain S , Shah AN , Santa‐Maria CA , Siziopikou K , Rademaker A , Helenowski I , et al. Phase I study of alpelisib (BYL‐719) and trastuzumab emtansine (T‐DM1) in HER2‐positive metastatic breast cancer (MBC) after trastuzumab and taxane therapy. Breast Cancer Res Treat. 2018;171(2):371–81. 10.1007/s10549-018-4792-0 29850984

[92] Saura C , Hlauschek D , Oliveira M , Zardavas D , Jallitsch‐Halper A , de la Peña L , et al. Neoadjuvant letrozole plus taselisib versus letrozole plus placebo in postmenopausal women with oestrogen receptor‐positive, HER2‐negative, early‐stage breast cancer (LORELEI): a multicentre, randomised, double‐blind, placebo‐controlled, phase 2 trial. Lancet Oncol. 2019;20(9):1226–38. 10.1016/S1470-2045(19)30334-1 31402321

[93] Infinity Pharmaceuticals presents updated data from phase 2 MARIO‐275 trial in urothelial cancer (UC) and phase 2 MARIO‐3 trial in triple negative breast cancer (TNBC) . News release. Infinity Pharmaceuticals. 2021 July 27 [cited 2021 July 28]. Available from: https://investors.infi.com/node/17446

[94] García‐Sáenz JÁ , Martínez‐Jáñez N , Cubedo R , Jerez Y , Lahuerta A , González‐Santiago S , et al. Sapanisertib plus fulvestrant in postmenopausal women with estrogen receptor‐positive/HER2‐negative advanced breast cancer after progression on aromatase inhibitor. Clin Cancer Res. 2022;28(6):1107–16. 10.1158/1078-0432.CCR-21-2652 34980598 PMC9365359

[95] Schmid P , Zaiss M , Harper‐Wynne C , Ferreira M , Dubey S , Chan S , et al. Fulvestrant plus vistusertib vs fulvestrant plus everolimus vs fulvestrant alone for women with hormone receptor‐positive metastatic breast cancer: the MANTA phase 2 randomized clinical trial. JAMA Oncol. 2019;5(11):1556–64. 10.1001/jamaoncol.2019.2526 31465093 PMC6865233

[96] Study of LEE011 , BYL719 and letrozole in advanced ER+breast cancer. [EB/OL]. (2014‐05‐20) [2022‐05‐10]. https://www.mycancergenome.org/content/clinical_trials/NCT01872260

[97] Sharma P , Farooki A , Fasching PA , Klauss G , Wong C , André F , et al. EPIK‐B3: a phase III, randomised, double‐blind (DB), placebo (PBO)‐controlled study of alpelisib (ALP) + nab‐paclitaxel (nab‐PTX) in advanced triple‐negative breast cancer (TNBC) with either PIK3CA mutation or phosphatase and tensin homolog (PTEN) loss without PIK3CA mutation. 2020 ESMO Poster 349 TiP.

[98] Hurvitz SA , Chia SKL , Ciruelos EM , Hu X , Im S , Janni M , et al. EPIK‐B2: a phase III study of alpelisib (ALP) as maintenance therapy with trastuzumab (T) and pertuzumab (P) in patients (pts) with PIK3CA‐mutated (mut) human epidermal growth factor receptor‐2–positive (HER2+) advanced breast cancer (ABC). 2020. ESMO Poster 352 TiP.

[99] Hurvitz SA , Andre F , Jiang Z , Shao Z , Mano MS , Neciosup SP , et al. Combination of everolimus with trastuzumab plus paclitaxel as first‐line treatment for patients with HER2‐positive advanced breast cancer (BOLERO‐1): a phase 3, randomised, double‐blind, multicentre trial. Lancet Oncol. 2015;16(7):816–29. 10.1016/S1470-2045(15)00051-0 26092818

[100] André F , O'Regan R , Ozguroglu M , Toi M , Xu B , Jerusalem G , et al. Everolimus for women with trastuzumab‐resistant, HER2‐positive, advanced breast cancer (BOLERO‐3): a randomised, double‐blind, placebo‐controlled phase 3 trial. Lancet Oncol. 2014;15(6):580–91. 10.1016/S1470-2045(14)70138-X 24742739

